# Copy number loss Of APP cause thoracic aortic dissection

**DOI:** 10.1038/s41440-025-02315-8

**Published:** 2025-08-07

**Authors:** Qiannan Gao, Minghui Bao, Jiangshan Tan, Haizeng Zhang, Luyun Fan, Yiran Zhou, Liyan Mao, Chengjun Huang, Weili Zhang, Bin Geng, Xiaohan Fan, Jun Cai, Zhenzhen Chen

**Affiliations:** 1 Beijing Anzhen Hospital of Capital Medical University and Beijing Institute of Heart Lung and Blood Vessel Diseases, Beijing, China; 2 Fuwai Hospital, National Center for Cardiovascular Diseases of China, CAMS and PUMC, Beijing, China; 3 Department of Cardiology, Peking University First Hospital, Peking University, Beijing, China; 4 Department of Biomedical Informatics, Department of Physiology and Pathophysiology, Center for Noncoding RNA Medicine, MOE Key Lab of Cardiovascular Sciences, School of Basic Medical Sciences, Peking University, Beijing, China

**Keywords:** Aortic dissection, Whole genome sequencing, Copy number variants, APP, Vascular smooth muscle cell.

## Abstract

Thoracic aortic dissection (TAD) is a leading cause of sudden cardiovascular death. Although a limited number of copy number variations (CNVs) have been reported in small cohorts of patients with hereditary TAD or sporadic aortic dissection, a comprehensive investigation and functional validation of CNVs in sporadic TAD using large-scale whole genome sequencing (WGS) data remain lacking. To address this gap, we conducted whole genome sequencing in two independent case–control studies, involving 257 patients with sporadic TAD and 132 controls. We generated gene knockout mice to explore the role of the target gene in TAD progression in vivo. Additionally, RNA-seq analysis and molecular biology experiments were performed in vitro to elucidate the underlying mechanisms. In the discovery and validation cohorts, we identified four CNVs genes *(DSCAM*, *APP*, *LINC00907*, *PROCR*) potentially pivotal in the pathogenesis of TAD. Among these, only APP displayed reduced expression in the aortas of TAD patients compared to controls. Deletion of APP exacerbated elastic fiber fragmentation and promoted TAD formation in both β-aminopropionitrile (BAPN)-induced and PCSK9/AngII-induced TAD models. In vitro, the loss of APP facilitated vascular smooth muscle cells (VSMCs) apoptosis and the switch to a secretory phenotype. Our study is the first to report novel CNVs of APP in TAD, demonstrating that APP deficiency accelerates the initiation and progression of TAD. These findings suggest that APP represents a promising therapeutic target and a potential genetic risk factor for TAD.

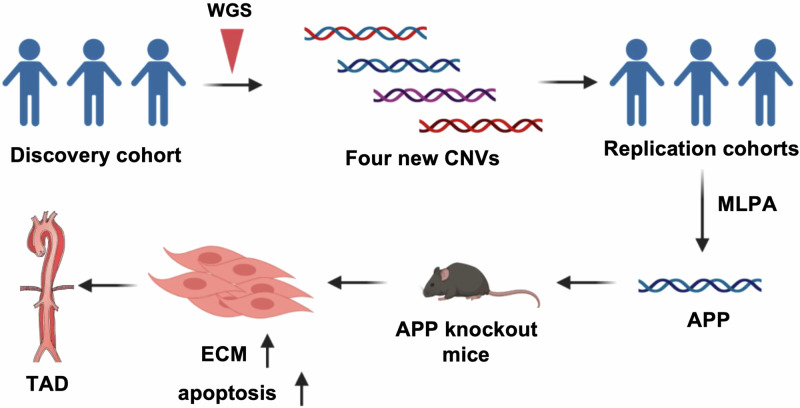

## Introduction

Aortic dissection holds a position among the most severe cardiovascular disorders, characterized by an astonishing rate of morbidity and mortality, and its incidence is experiencing an alarming increase [1]. The classification of aortic dissection mainly includes two types: Type A and Type B. Type A aortic dissection involves the ascending aorta, while Type B is confined to the descending aorta [2, 3]. Prompt diagnosis is of paramount importance as aortic dissection can progress rapidly and give rise to complications such as organ ischemia, heart failure, or rupture of the aorta, which is frequently fatal [4]. An expanding corpus of research emphasizes the crucial role of genetic deviations in the initiation and development of thoracic aortic dissection (TAD) [5, 6]. Approximately 20% of individuals with thoracic aortic aneurysms or dissections display an autosomal dominant pattern of inheritance of the condition within the family, signifying that the disease results from a mutation in a single gene. Genetic variants that predispose individuals to aortic diseases tend to occur in genes implicated in the structure and function of the aortic wall, specifically the extracellular matrix, TGF-β pathway, and smooth muscle cell contractile. For instance, genomic aberrations in chromosomal regions such as 15p21 [7] and 16q12.2-13.13 [8], along with genes like *FBN1* [9], *MYH11* [10], *ACTA2* [11], *TGFB2* [12], *COL3A1* [13], *SMAD3* [14] and *LOX* [15], are discerned in familial and syndromic TAD. Nevertheless, research is scarce on sporadic TAD, leaving the genetic underpinnings of such cases largely unexplored.

Copy number variations (CNVs), characterized by the amplification or reduction of chromosomal segments, modulate gene expression through dosage effects and have been implicated in numerous disease [16, 17]. Utilizing whole-genome sequencing (WGS) technology, which provides a comprehensive scan of an organism’s genome through high-throughput sequencing, has proven highly valuable in identifying CNVs in numerous studies [18]. Furthermore, compared to genotyping arrays, WGS offers a more comprehensive assessment of CNVs and other genomic structural alterations [19, 20]. Regrettably, few studies have probed into the mutational landscape, encompassing CNVs and the associated genes, in the domain of sporadic TAD.

In the current study, we investigated the CNVs of sporadic TAD using WGS. Intriguingly, we identified a novel CNV associated with the amyloid beta precursor protein (APP) gene in cohorts of TAD patients. Subsequently, we generated APP global knockout mice and different mouse TAD models to explore its role in the progression of TAD. Additionally, we unveiled the molecular mechanisms of APP in VSMCs. Our findings indicate that APP deficiency aggravates TAD progression, suggesting that APP might be an inherent predisposing gene for TAD and a potential candidate for genetic screening in TAD populations.

## Methods

A detailed description of all methods can be found in the supplementary material.

### Patient cohorts and study design

Individuals with sporadic non-familial TAD were recruited from outpatient, emergency, and inpatient departments of Fuwai Hospital and Chaoyang Hospital in Beijing, China, and were included as the discovery cohort. Individuals with syndromic dissection (including Marfan syndrome, Loeys-Dietz syndrome, Ehlers-Danlos syndrome and other types of syndromic dissection) or a family history of thoracic aortic aneurysms and dissections or who were under the age of 30 years were excluded. After the exclusion of patients, 100 patients with TAD were included as the discovery cohort. A total of 132 control subjects were included from participants who underwent health examinations in Chaoyang Hospital. Clinical information and blood samples were collected by trained clinical doctors after the approval of the study by the ethics committees of both hospitals. Informed consent was obtained from all participants.

**Fig. 1:**
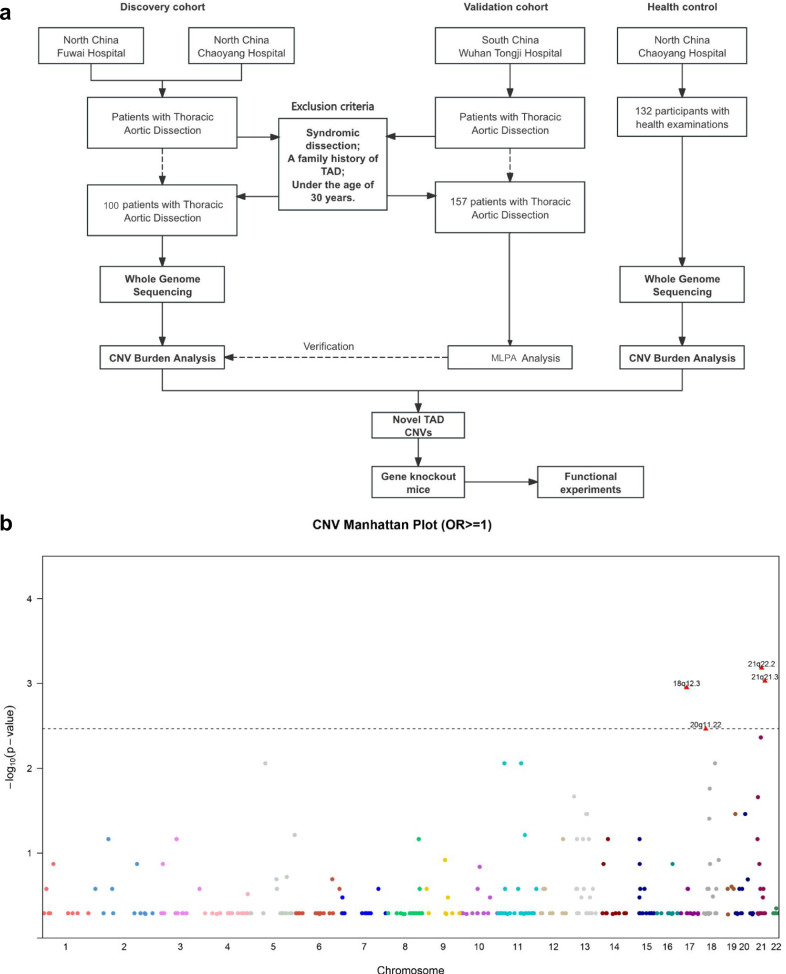
Flowchart of study design and the CNV association study. **a** The overall flowchart of the research. **b** A Manhattan plot of discovery stage genome-wide association results from the comparison of TAD cases to healthy controls. For each tested CNV, the significance is displayed on the y-axis as the -log10 of the *P*-value. The -log10 results are ordered along the x-axis by chromosome, with each colored dot representing a different CNV. CNVs located in the same chromosome are presented with the same color. TAD, Thoracic aortic dissection; CNVs, copy number variants

The participants of the replication cohorts were recruited from the outpatient, emergency, and inpatient departments of Wuhan Tongji Hospital, Hubei, China. Clinical information and blood samples were collected by trained clinical doctors upon obtaining approval from the ethics committees of the hospital. Informed consent was obtained from all participants (Fig. 1a).

The existence of aortic aneurysm was recorded before surgery through echocardiography or computed tomography/magnetic resonance imaging. The clinical diagnosis was determined before surgery by expert clinicians. A diagnosis of Marfan syndrome was made in accordance with the revised Ghent criteria, and a diagnosis of non-syndromal TAD was made if the patient was <60 years of age, with any other predisposing conditions, such as hypertension or atherosclerosis, and no family history. The clinical characteristics of the discovery and validation cohorts are presented in Table 1.

**Table 1:** Clinical Characteristics of the Discovery and Replication Cohorts

Variable	Controls (*N* = 132)	Discovery Cohort			Replication Cohort		
		Type A (*N* = 100)	X^2^/t	*p*	Type A (*N* = 157)	X^2^/t	*p*
**Age (years)**	53.7 ± 9.5	52.7 ± 12.3	−0.699	0.485	51.2 ± 12.4	1.895	0.059
**Male,** ***n*** **(%)**	78 (59.0)	62 (62.0)	0.201	0.654	96 (70.1)	3.798	0.051
**BMI (kg/m2)**	24.7 ± 4.2	25.2 ± 3.7	0.945	0.346	25.2 ± 3.9	−1.048	0.295
**Smoking (%)**	52 (39.4)	34 (34.0)	0.710	0.400	75 (47.8)	2.043	0.153
**Hypertension (%)**	73 (55.6)	65 (65.0)	2.220	0.135	67 (42.3)	4.578	0.032
**Diabetes (%)**	3 (2.3)	4 (4.0)	0.580	0.468	5 (3.2)	0.222	0.638

Variables were described by mean ± standard deviation when applicable. Age, age of inclusion; BMI, body mass index; Smoking, current smoker; Hypertension, diagnosed with hypertension or taking antihypertensive therapy; Diabetes, diagnosed with diabetes or taking hypoglycemic agents

### Whole-genome sequencing

DNA of all participants was screened for CNVs using Illumina platform. The clustering of the index-coded samples was conducted on a cBot Cluster Generation System using Hiseq X HD PE Cluster Kit (Illumina) in accordance with the manufacturer’s instructions. The raw image files obtained from Hiseq X were processed with Illumina pipeline for base calling. Filter reads with adapter contamination (>10 nucleotide aligned to the adapter, allowing≤10% mismatches). Valid sequencing data were mapped to the reference genome (UCSC hg19) by Burrows-Wheeler Aligner (BWA) software (Li H et al. 2009-1) to obtain the original mapping result in BAM format. Samtools (Li H et al. 2009-2) and Picard (http://broadinstitute.github.io/picard) were utilized to sort bam files and perform duplicate-marking to generate final bam files. Samtools mpileup and bcftools were employed for variant calling and identifying SNPs and indels.

CNV refers to the increase or reduction of copy number of large fragments in the genome. There are two types of CNV: deletion and duplication. Since CNV detection is not so highly accurate, we utilized the reliable, user-friendly computational pipeline-Control-FREEC (Boeva V et al. 2012) to discover disruptive genic CNVs in human genetic studies of disease, which might be overlooked by standard approaches. ANNOVAR (Wang K et al. 2010) was carried out for annotation for Variant Call Format files obtained in the previous step. Variants obtained from previous steps were then filtered with the MAF > 1% in the 1000 Genomes databases (1000 Genomes Project Consortium). Finally, the retained nonsynonymous SNVs were submitted to PolyPhen-2 (Adzhubei I et al. 2013), SIFT (Ng PC et al. 2003), Mutation Taster (Schwarz J M et al. 2010) and CADD (Martin K et al. 2014) for functional prediction. If at least half of the four software indicated that the SNV was not benign, it was retained.

### Statistical analysis

Statistical analysis was conducted with R (RNA-seq) and GraphPad Prism 8.0. Unless otherwise indicated, values are presented as mean ± standard error of the mean (SEM). All data were tested for normality and equal variance. If the data passed these tests, Student’s t-test was carried out for the comparison of two groups. If the data did not pass these tests, the Mann-Whitney U test was employed to compare 2 groups. Fisher exact test was utilized for the comparison of the incidence of BAPN-induced and PCSK9/AngII-induced TAD. *P* < 0.05 was considered statistically significant.

## Results

### Clinical characteristics of the discovery and validation cohorts

The workflow is presented in Fig. 1a. A total of 389 participants were included in this study. The discovery cohort included 100 sporadic TAD cases and 132 control subjects (Fig. 1a and Table 1). These individuals were matched regarding ethnicity, age, sex, body mass index (BMI), the prevalence of diabetes, hypertension, and cigarette smoking history. Approximately 62% of the overall cohort was male. Within the TAD cohort, the median age at onset approximated 52.7 years, with hypertension manifesting in about 65% of these cases. For the validation cohort, we recruited 157 patients with TAD, with no significant differences in the baseline characteristics except for the prevalence of hypertension (Table 1).

### WGS of type A TAD cases and controls

WGS was carried out on blood samples from TAD cases and controls in the discovery cohort. The comprehensive genomic data analysis for the 100 individuals diagnosed with sporadic type A aortic dissections unveiled a total of 21,479 CNVs (Supplementary Fig. 1a). The majority of these CNVs ranged between 2 kb and 10 kb in size (Supplementary Fig. 1b), with data revealing 19.90 deletions and 23.77 duplications per genome within coding sequences (Supplementary Fig. 1a). These variations were annotated across multiple databases to identify pathogenic CNVs relevant to human diseases (Supplementary Fig. 1c).

### CNVs associated with type A TAD in the discovery and validation cohorts

CNV burden analysis identified a total of 433 CNVs and among them, three amplifications and a single deletion occurred significantly more prevalently (*P* < 0.05, Supplementary Table. 1) in patients with sporadic TAD when compared to control participants (Fig. 1b and Table 2). Interestingly, no CNVs were found to exert a protective effect in relation to TAD (Supplementary Fig. 1d). The CNVs with the most significance associations were localized to four genes, namely *DSCAM* (*P* < 0.001), *APP* (*P* = 0.004), *LINC00907* (*P* = 0.016), and *PROCR* (*P* = 0.033). An intriguing convergence of copy number augmentations in both *DSCAM* and *APP* was discerned in a quartet of the TAD cases, amounting to 4% (Supplementary Table 2).

**Table 2:** Newly Identified CNVs in Discovery Cohort and CNV Burden Analysis in Replication Cohorts

Gene	Chr.	Control			Discovery Cohort			Replication Cohort		
		Loss	Gain	Total	Loss	Gain	Total	Loss	Gain	Total
APP*	21	0	0	0	8	0	8	26	6	32
DSCAM*	21	1	0	1	13	0	13	20	17	37
LINC00907*	18	0	0	0	6	0	6	24	7	31
PROCR*	20	0	0	0	0	5	5	13	14	27

Gene, gene that the CNV was located, *Chr* chromosome, *TA* type A aortic dissection, *CON* control, *Loss* copy number loss, *Gain* copy number gain, *Burden* total CNV burdens calculated by combining copy number gains and losses. ^*^p < 0.05

To confirm the pathogenic roles of the four identified CNVs, we conducted multiplex ligation-dependent probe amplification (MLPA) analyses in validation cohorts (Table 1), the result indicated that there are significantly greater CNV burdens in the four tested genes (*DSCAM*, *APP*, *LINC00907*, and *PROCR*) compared with the controls (Table 2).

### APP expression is reduced in human aortic dissection lesions

**Fig. 2:**
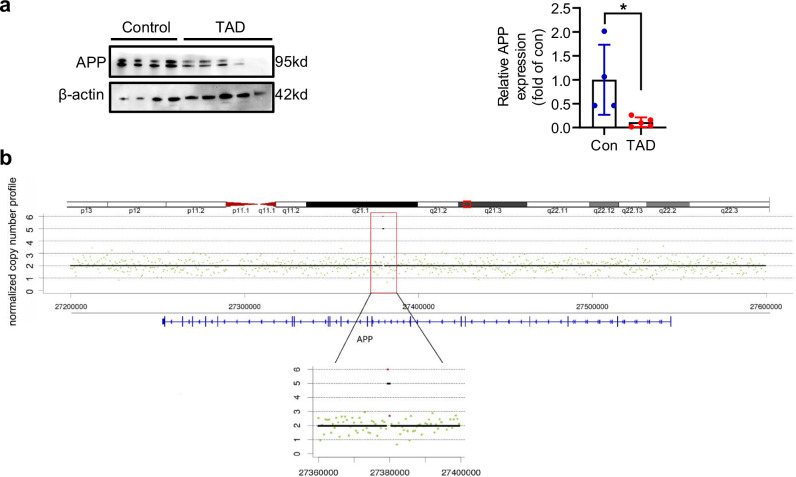
APP expression is reduced in human and mouse aortic dissection tissues. **a** Expression of APP protein in the aorta of TAD patients and healthy individuals was determined using western blot. The left pannel is representative image, the right pannel is statistical graph. **b** The schematic diagram of CNVs of APP. Detailed position of the 21q21.3 deletion. The known protein-encoding gene affected by the deletion, APP, is represented by the blue bars beneath the chromosome. The x-axis represents the CNV location on chromosomes and the y-axis indicates the normalized copy number profile. The black dots are normalized copy numbers. Data were presented as Mean±S.E.M. *P < 0.05. APP, amyloid beta precursor protein

Subsequently, we analyzed the protein expression levels of the four candidate genes in aortic dissection specimens collected from four sporadic TAD patients and compared them with specimens from five healthy individuals. Only APP’s expression demonstrated significant reduction within aortic extracts from TAD patients when compared to those in healthy counterparts (Fig. 2a and Supplementary Fig. 2a, b). A schematic diagram of the CNVs of *APP* is shown in Fig. 2b. In summary, our findings imply a decreased expression of APP in aortic dissection specimens derived from human TAD subjects.

### APP deficiency augments the onset of TAD

**Fig. 3:**
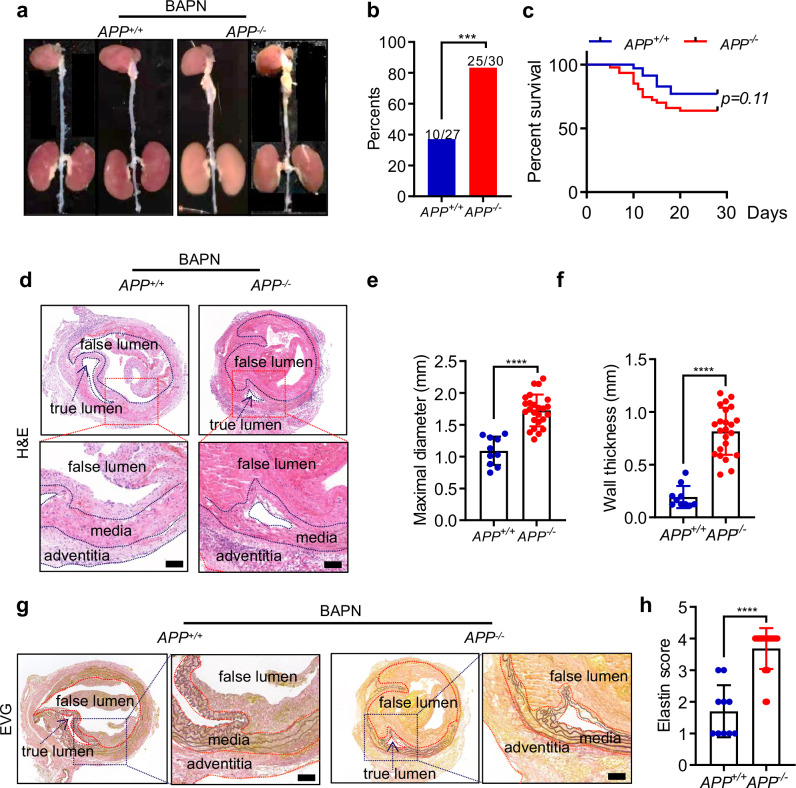
APP knockout promoted the development of TAD in the BAPN-induced mice. In the BAPN-induced TAD model, **a** Representative images of formation of TAD in thoracic aorta in APP^−/−^ mice and APP^+/+^ mice. **b**, **c** TAD incidence and survival curve of APP^−/−^ mice (*n* = 47) and APP^+/+^ mice (*n* = 35). **d** Representative images of H&E staining in the aorta of APP^+/+^ mice and APP^−/−^ mice. The media, adventitia, and false lumen regions are outlined with black dashed lines for enhanced visualization, aiding in the clear distinction of the structural changes associated with aortic dissection. **e**, **f** The maximal diameter and wall thickness in thoracic aorta in APP^−/−^ mice and APP^+/+^ mice. **g** Representative images of EVG staining demonstrating elastin fiber degeneration in the thoracic aorta of APP^+/+^ mice and APP^−/−^ mice. The media, adventitia, true lumen, and false lumen regions are outlined with red dashed lines. **h** Elastin score show the degree of elastin degradation in thoracic aorta in APP^−/−^ mice and APP^+/+^ mice. Data were presented as Mean±S.E.M. ***P < 0.001, ****P < 0.0001. Scale bar = 200 µm

To explore the role of APP in the TAD progression, *APP*-deficient mice were constructed by CRISPR/Cas9 methodology (Supplementary Fig. 3a). The efficacy of *APP* ablation was confirmed by western blot analysis (Supplementary Fig. 3b). β-aminopropionitrile (BAPN), acting as a lysyl oxidase antagonist, curtails the cross-linking between elastin and collagen, thereby instigating the onset of aortic dissection [21, 22]. We treated 3-week-old *APP*^*−/−*^ mice and wild-type littermates (*APP*^*+/+*^) with BAPN (1 g/kg/day) in drinking water for 4 weeks to induce the mice TAD model (Fig. 3a). Although there was no statistically significant difference in mortality, the TAD occurrence soared to 83.3% in the *APP*^*−/−*^ group, while only 37.0% in the *APP*^*+/+*^ mice (Fig. 3b, c) Moreover, hematoxylin and eosin (H&E) staining revealed that APP knockout augmented aortic diameter and wall thickness (Fig. 3d–f).The extent of elastin deterioration was appraised via Elastin-Van Gieson’s (EVG) staining, with evaluations as delineated in the methods [23] (Fig. 3e and Supplementary Fig. 3c). The results showed that the elastin score was higher in the *APP*^*−/−*^ mice than that in the *APP*^*+/+*^ mice (Fig. 3g, h). Collectively, these data suggest that APP deficiency aggravates TAD formation, enhances elastin disruption and degradation in the BAPN-induced TAD model.

**Fig. 4:**
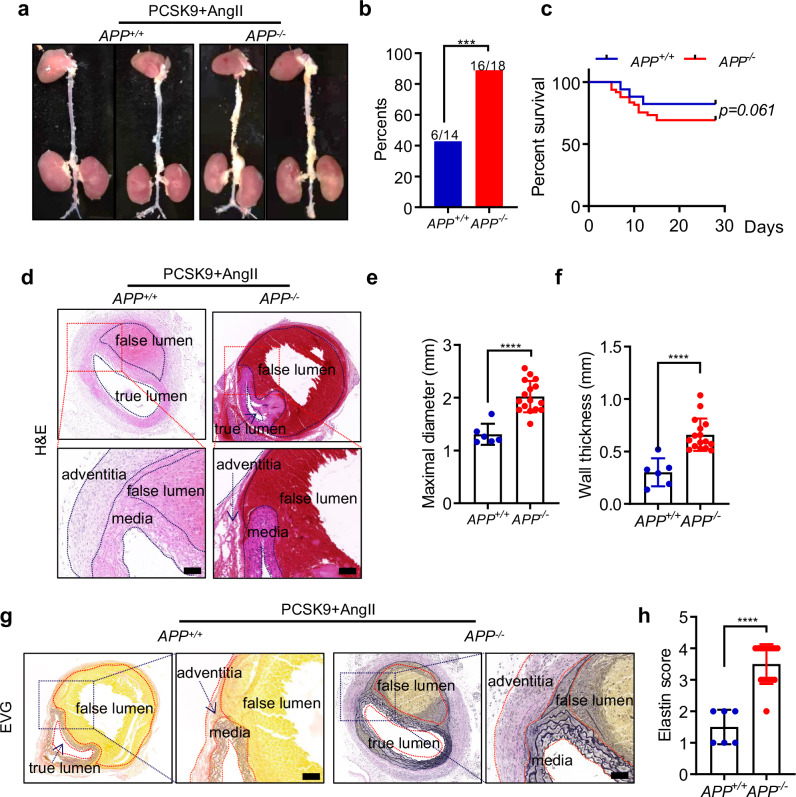
APP knockout promoted the development of TAD in the PCSK9/AngII mice. In the PCSK9/AngII-induced TAD model, **a** Representative images of formation of TAD in thoracic aorta in APP^−/−^ mice and APP^+/+^ mice. **b**, **c** TAD incidence (**b**) and survival curve (**c**) of APP^−/−^ mice (*n* = 33) and APP^+/+^ mice (*n* = 17). **d** Representative images of H&E staining in the aorta of APP^+/+^ mice and APP^−/−^ mice. The media, adventitia, true lumen, and false lumen regions are outlined with black dashed lines for enhanced visualization, aiding in the clear distinction of the structural changes associated with aortic dissection. **e**, **f** The maximal diameter and wall thickness in thoracic aorta in APP^−/−^ mice and APP^+/+^ mice. **g** Representative images of EVG staining demonstrating elastin fiber degeneration in the thoracic aorta of APP^+/+^ mice and APP^−/−^ mice. The media, adventitia, ture lumen, and false lumen regions are outlined with red dashed lines. **h** Elastin score show the degree of elastin degradation in thoracic aorta in APP^−/−^ mice and APP^+/+^ mice. Data were presented as Mean±S.E.M. ***P < 0.001, ****P < 0.0001. Scale bar = 200 µm

To confirm the crucial role of APP in TAD, we established another TAD mouse model by PCSK9 overexpression (a single tail vein injection of adeno-associated virus rAAV8-HCRApoE/hAAT-D377Y-mPCSK9) combined with a western diet two weeks prior to AngII infusion (PCSK9/AngII-induced TAD mouse model) (Fig. 4a). Consistent with observations from the BAPN-induced model, APP knockout increased the incidence of TAD, augment aorta’s maximal diameter, aggravated elastin disruption and degradation (Fig. 4b–g). Collectively, these findings demonstrate that APP deficiency promotes the development of TAD.

### Loss of APP promoted VSMCs apoptosis and collagen accumulation in vivo

**Fig. 5:**
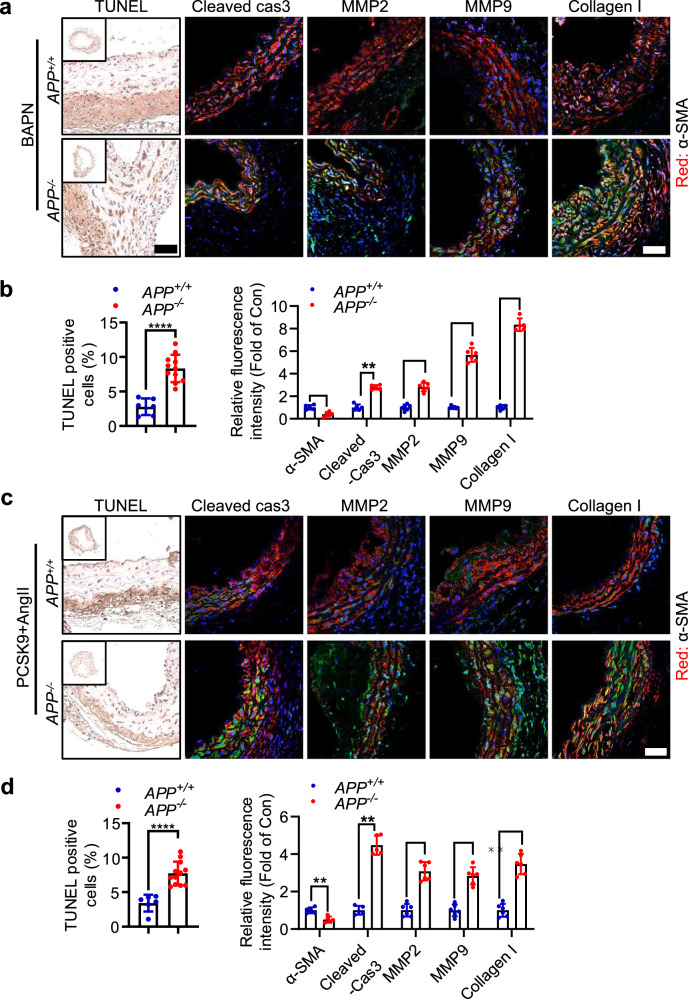
**a** APP knockout promoted VSMCs apoptosis and enhanced secretory phenotypic VSMCs in the aorta of TAD models. **b** Percentage of TUNEL-positive nuclei in the aortic media of BAPN-induced TAD model was examined using TUNEL staining. Normal cell nuclei are blue and apoptotic cell nuclei are brown. Expression of α-SMA, cleaved caspase3, MMP2, MMP9 and Collagen I in the aorta wall of BAPN-induced TAD model was examined using immunostaining analysis. **c**, **d** Percentage of TUNEL-positive nuclei in the aortic media of PCSK9/AngIIII-induced TAD model was examined using TUNEL staining. Expression of α-SMA, cleaved caspase3, MMP2, MMP9 and Collagen I in the aorta wall of PCSK9/AngII-induced TAD model was examined using Immunostaining analysis. Data were presented as Mean±S.E.M. ****P < 0.0001. Scale bar = 50 µm. TUNEL, Terminal deoxynucleotidyl transferase dUTP nick end labeling

Pivotal characteristics of TAD encompass smooth muscle cells (SMCs) depletion, VSMCs switch from contractile phenotype to a secretory/inflammatory phenotype, and extracellular matrix degradation [24]. Subsequent investigations focused on the influence of *APP* on VSMCs. Within the BAPN-induced TAD model, loss of *APP* increased the presence of cleaved caspase 3 and the proportion of TUNEL-positive cells by immunostaining (Fig. 5a, b). In addition, *APP*^*−/−*^ mice exhibited higher expression of MMP2, MMP9, and collagen I in the aortic tissues compared to *APP*^*+/+*^ mice (Fig. 5a, b). Consistently, APP deficiency promotes VSMCs apoptosis and extracellular matrix degradation in the PCSK9/AngII-induced TAD mouse model (Fig. 5c, d).

### APP deficiency aggravated VSMCs apoptosis and switch to secretory phenotype in vitro

**Fig. 6:**
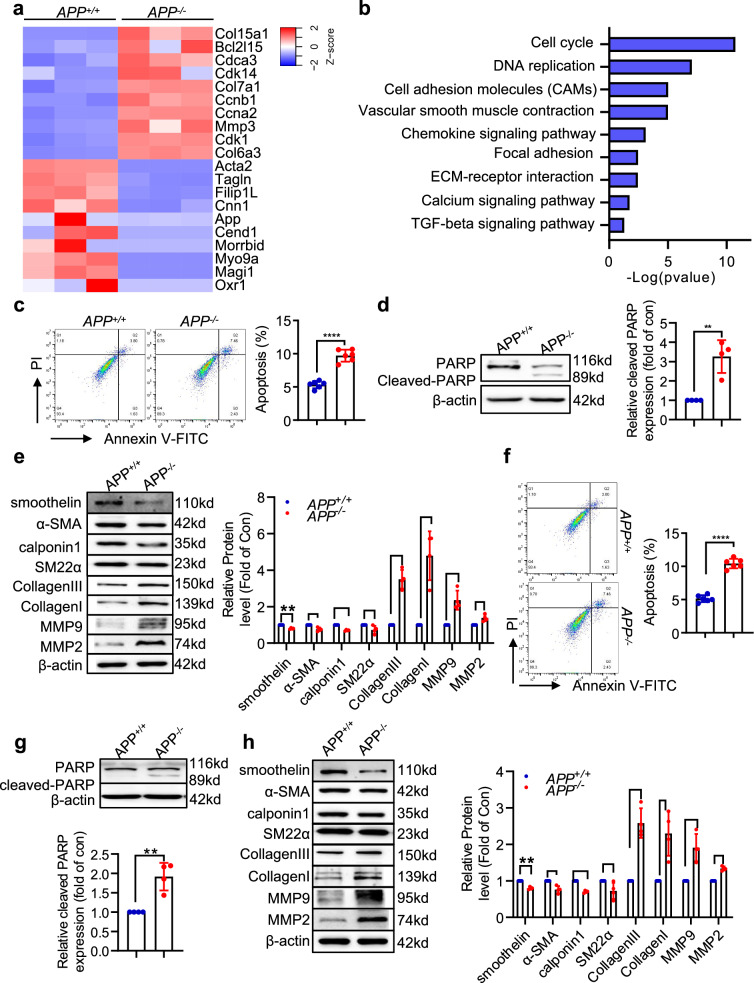
APP knockout promoted apoptosis and secretory phenotypes of MASMCs. Primary MASMCs were isolated from the APP−/− mice and WT littermates. **a**, **b** Heatmap (**a**) and KEGG (**b**) analysis of differentially expressed genes in primary MASMCs treated with AngII (1 μmol/L) plus ox-LDL (150 ng/ml) for 24 h. **c** Cell apoptosis in MASMCs with BAPN (1 mmol/L) treatment was examined using Annexin V/ PI staining and flow cytometry analysis. **d** Expression of cleaved PARP in MASMCs with BAPN treatment was examined using western blot. **e** Expression of contractile markers including SM22α, calponin-1, α-SMA, smoothelin and secretory markers including MMP2, MMP9, Collagen I and Collagen III in MASMCs with BAPN treatment were examined using western blot. The left pannel is representative image, the right pannel is statistical graph. **f**–**j** Cell apoptosis in MASMCs treated with AngII plus ox-LDL was examined using Annexin V/PI staining and flow cytometry analysis (**f**) and western blot (**j**). **h** Expression of contractile markers and secretory markers in MASMCs with AngII plus ox-LDL treatment were examined using western blot. Data were presented as Mean±S.E.M. *P < 0.05, **P < 0.01, ***P < 0.001, ****P < 0.0001. Scale bar = 100 µm. MASMCs, Mouse aortic smooth muscle cells, PARP, poly (ADP-ribose) polymerase

To comprehensively examine the function of *APP* in VSMCs, we performed RNA sequencing after primary murine aortic smooth muscle cells (MASMCs) from APP^−/−^ mice were exposed to AngII and ox-LDL for 24 h. Differential expression analysis showed that differential genes between *APP*^*−/−*^ and *APP*^*+/+*^ are enriched in the cell cycle, vascular smooth muscle contraction, and extracellular matrix signaling pathway (Fig. 6a, b). For confirmation, we isolated primary MASMCs to investigate the effect of APP on VSMCs function. In the basal condition, there was no significant difference between *APP*^*−/−*^ and *APP*^*+/+*^ MASMCs regarding the expression of the apoptosis marker (cleaved PARP), VSMCS contractile markers (smoothelin, α-SMA, calponin1, SM22α) and extracellular matrix markers (MMP2, MMP9, collagen I and collagen III) (Supplementary Fig. 5a–d). Subsequently, the MASMCs underwent treatment with BAPN or a combination of AngII and oxidized LDL to mimic TAD in vivo. When exposed to BAPN, the absence of *APP* increased the proportion of apoptotic cells (Fig. 6c). Poly ADP-ribose polymerase (PARP) plays a crucial role in apoptosis by regulating DNA damage repair and energy metabolism, and its cleavage by caspases is a key indicator of the apoptotic process. APP deficiency promoted the production of cleaved PARP in contrast to the control (Fig. 6d). In line with VSMCs apoptosis, the expression of VSMCs contractile indicators (SM22α, α-SMA, calponin-1, and smoothelin) decreased in the *APP*-deficient group (Fig. 6e). Accordingly, MMP2, MMP9, Collagen I and collagen III underwent an augmentation in the APP-deficient group relative to their wild-type counterparts (Fig. 6e). Consistently, APP deficiency exacerbated VSMCs apoptosis and extracellular matrix degradation under AngII and oxidized-LDL stimulation (Fig. 6f, g).

Loss of copy number led to gene expression reduction. To better mimic the condition in vivo, we knocked down APP using siRNA (Supplementary Fig. 4). In line with APP deficiency, APP knockdown had no effect on the VSMCs apoptosis and extracellular matrix degradation (Supplementary Fig. 5b–f) in basal condition, but significantly augmented BAPN or a combination of AngII and ox-LDL induced VSMCs apoptosis and extracellular matrix degradation (Supplementary 6a-6h). All the above results suggested that APP loss aggravates VSMC apoptosis, VSMCs switch from contractile to secretory phenotype and extracellular matrix degradation after stimulus in vitro.

## Discussion

Aortic dissection presents a serious medical emergency with the imminent risk of rupture. Therefore, the crucial need for early detection and skilled intervention cannot be overemphasized. Our study utilized Whole Genome Sequencing (WGS) to investigate Copy Number Variation (CNV) burden in an initial discovery group, supplemented by validation cohorts. We identified that a novel CNV (copy number loss of APP) was interrelated with the onset of TAD. Furthermore, the absence of APP increased morbidity and mortality in the BAPN- and PCSK9/AngII-induced TAD murine models, and facilitated VSMCs apoptosis and phenotypic switch.

Genetics factors have been reported to play a critical role in the onset of aortic dissection. The non-familial, sporadic variant of thoracic dissection predominates, yet its genetic underpinnings remain unclear. CNVs, representing genomic structural oscillations characterized by either an augmentation or diminution of coding sequences [25], serve as pivotal conduits of genomic heterogeneity. These variations, as discovered, play instrumental roles in the genesis of myriad human maladies [26, 27]. While a scarcity of research has pivoted around the interplay of CNVs and aortic dissection, a handful of genes have been unveiled [28–30]. Prakash et.al. [29] identified 47 CNV locales in individuals plagued with thoracic aortic aneurysms and dissections. However, their results were limited to sequencing and network analysis, lack of verification of biological function in vivo.

In alignment with prior research, several CNVs unearthed in our analysis corroborate findings from earlier reports, such as CNVs of *FBN1* [9] and *MYH11* [10]. Remarkably, our research brought to light four hitherto undiscovered CNVs of genes (APP, DSCAM, PROCR, LINC00907) associated with the genesis of TAD. Among that, only the copy number loss of APP was subsequently confirmed in the validation cohorts. Further, we found that the APP protein level was decreased in the aorta of TAD patients. APP is a transmembrane protein that is cleaved through the activity of β and γ secretases [31]. The generated Aβ peptides are major components of amyloid plaques and are associated with Alzheimer’s disease pathogenesis [32]. Some studies have revealed that APP and its cleavage peptides play important roles in the pathogenesis of metabolic diseases, such as obesity and diabetes. Puig et al. found that a high-fat diet-induced obesity triggers pro-inflammatory changes in the brain and adipose tissue, marked by increased levels of APP contributing to the inflammation [33]. Another study indicated that increased Abeta in HFD-fed Tg2576 mice reduces brain-derived neurotrophic factor, leading to abnormal feeding behavior, increased food intake, obesity, and insulin resistance [34]. The role of APP in cardiovascular diseases, especially its involvement in the process of TAD occurrence, has not been reported. In our study, APP knockout accelerated the progression of TAD in BAPN- or PCSK9/AngII-induced models. Our results first clarified the essential role of APP in TAD.

Distinct from other cells, VSMCs evade terminal differentiation and are equipped for a physiological metamorphosis from the contractile to the “synthetic” phenotype when subjected to stimulus, producing a surplus of extracellular matrix (ECM) proteins, including collagens and MMPs [35, 36]. Quintessential characteristics of TAD encompass the progressive attrition of SMCs, a phenotypic transition from the contractile mode to a secretion-driven and inflammatory stance, and the erosion of the ECM [24]. Antecedent research illuminated that genetic aberrations in SMC-centric contractile genes, such as MYH1110, ACTA211, MYLK [37], facilitated disorder of the aortic wall’s structure, which contribute to aortic dissection. Similarly, APP depletion amplifies SMCs apoptosis in cerebral conduits, thereby aggravating cerebral amyloid angiopathy [38]. Additionally, APP gene transcription expression negatively correlated with MMP concentrations within cerebral substrates [39, 40]. In line with previous studies, our finding revealed that APP loss promoted VSMCs apoptosis, augmented VSMCs switch to secretory phenotype, enhanced collagen accumulation.

In summary, the present study revealed that loss of APP gene copy number stands as significant genetic determinants implicated in the onset of TAD. APP plays the essential role in TAD development and progression by regulating VSMCs apoptosis and phenotype switch. Our findings intimate that APP could potentially emerge as a novel therapeutic target for TAD intervention and a prospective candidate for genetic surveillance of the ailment.

## Supplementary information

Supplementary Figures

Supplementary Methods
