# Supplementary material for: Copy number loss Of APP cause thoracic aortic dissection
Source: Hypertens Res. 2025 Aug 7;48(10):2641–53. doi: 10.1038/s41440-025-02315-8 (PMC12497644; doi:10.1038/s41440-025-02315-8)

Supplementary Figures

Supplementary Figure 1

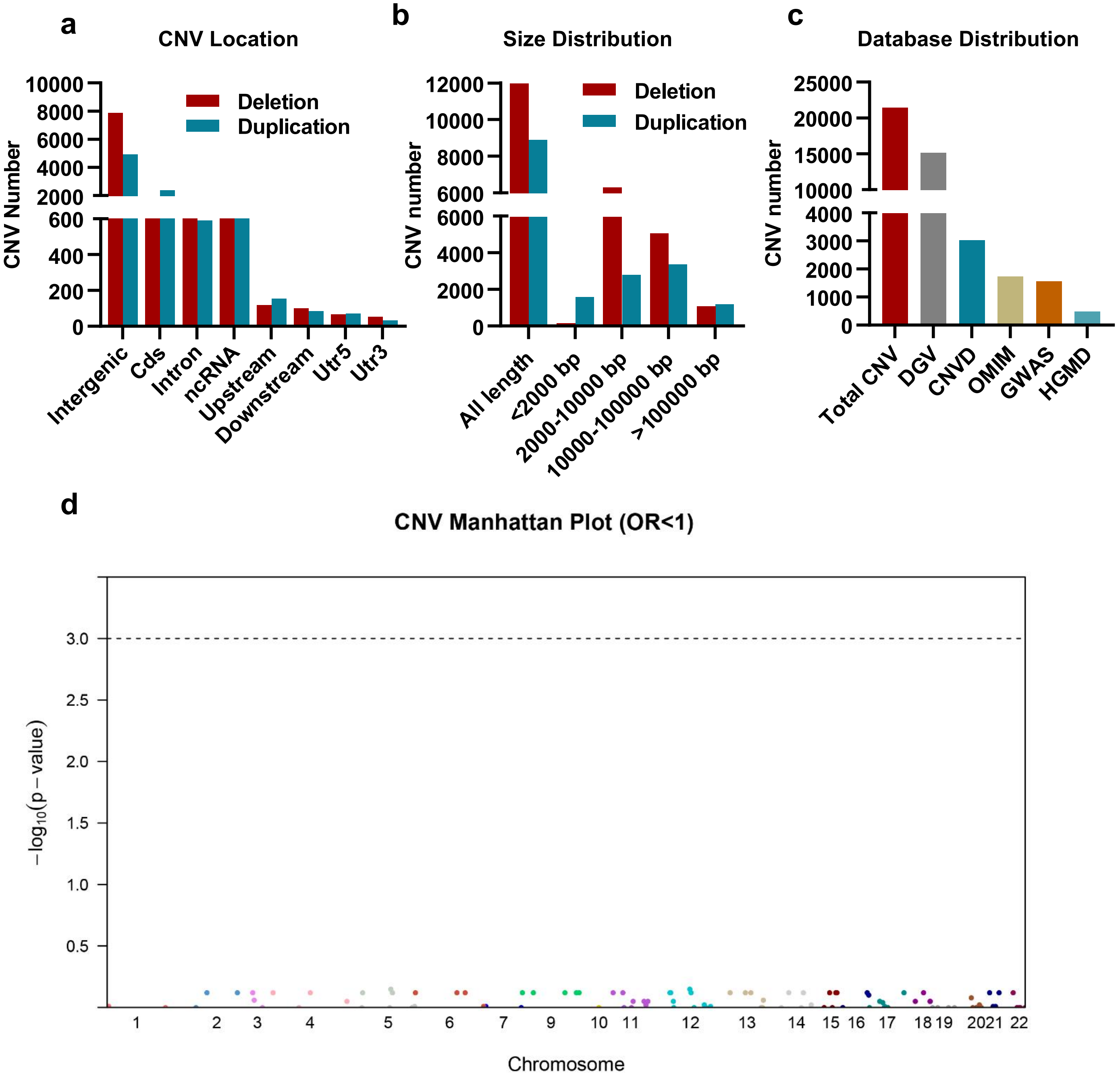

Supplementary Figure 2

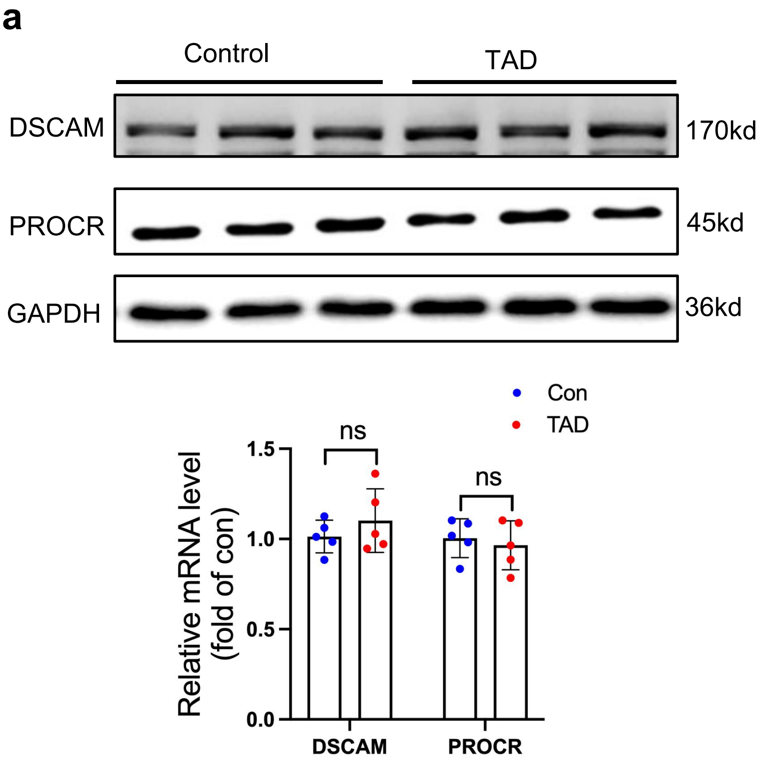

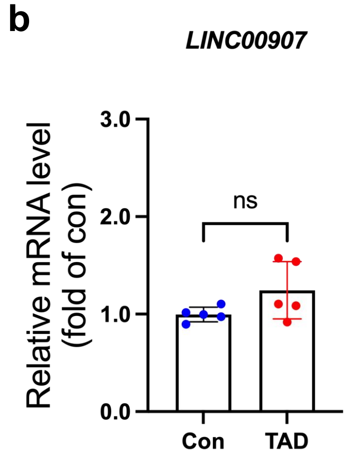

Supplementary Figure 3

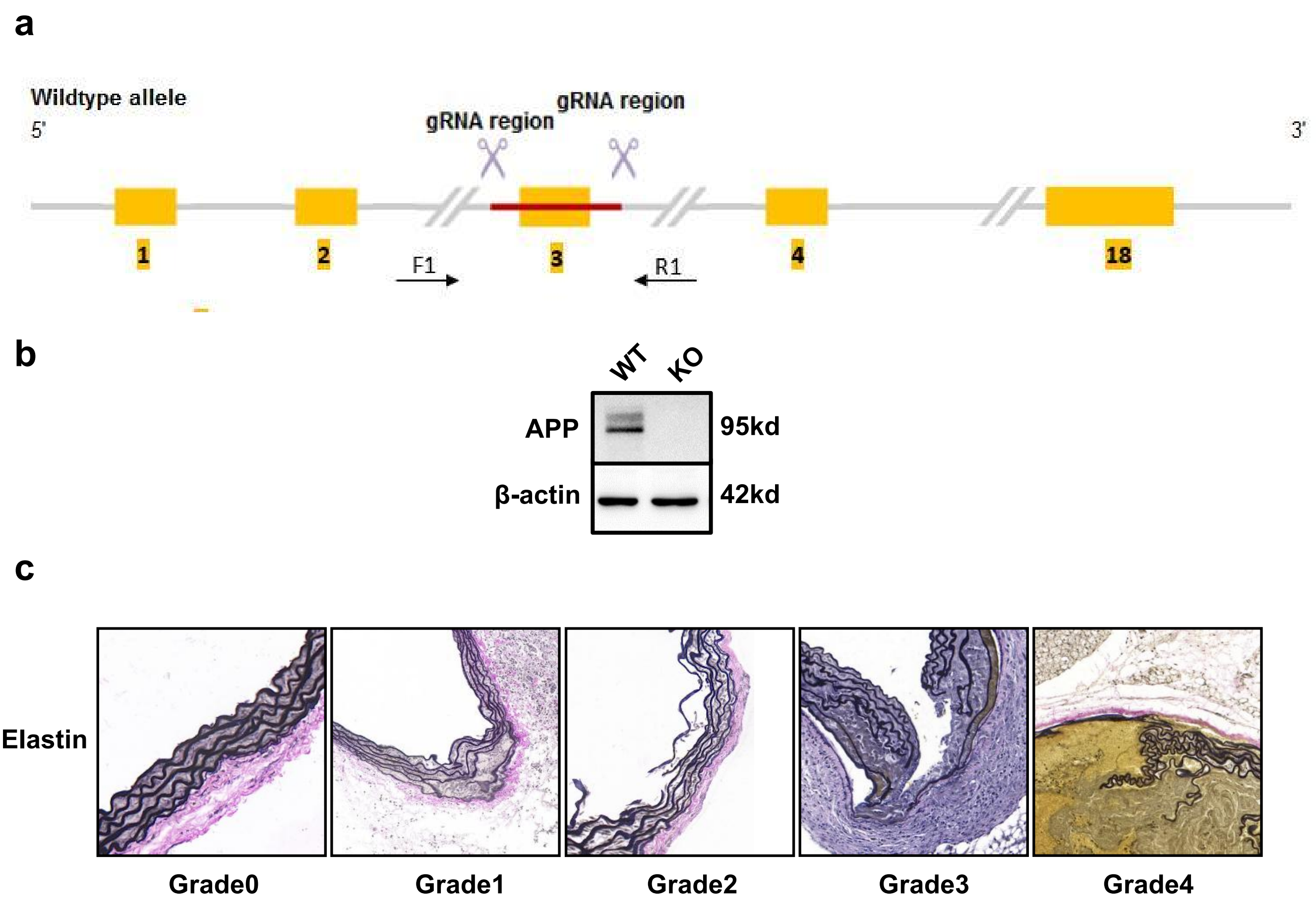

Supplementary Figure 4

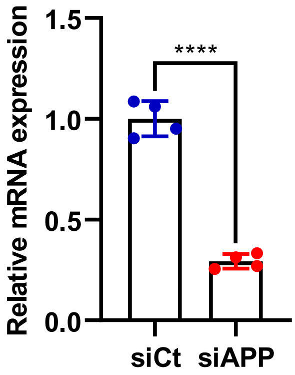

Supplementary Figure 5

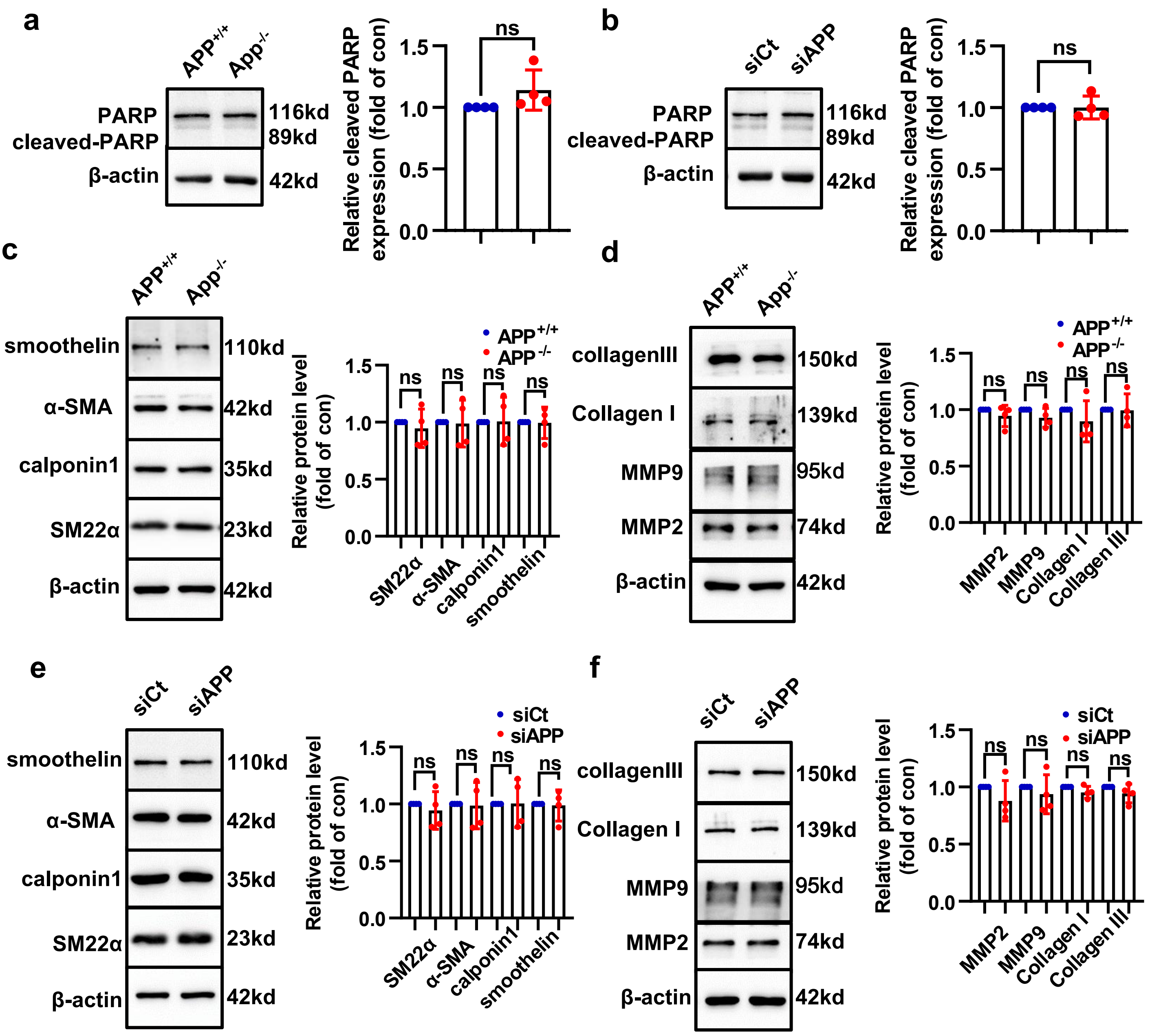

Supplementary Figure 6

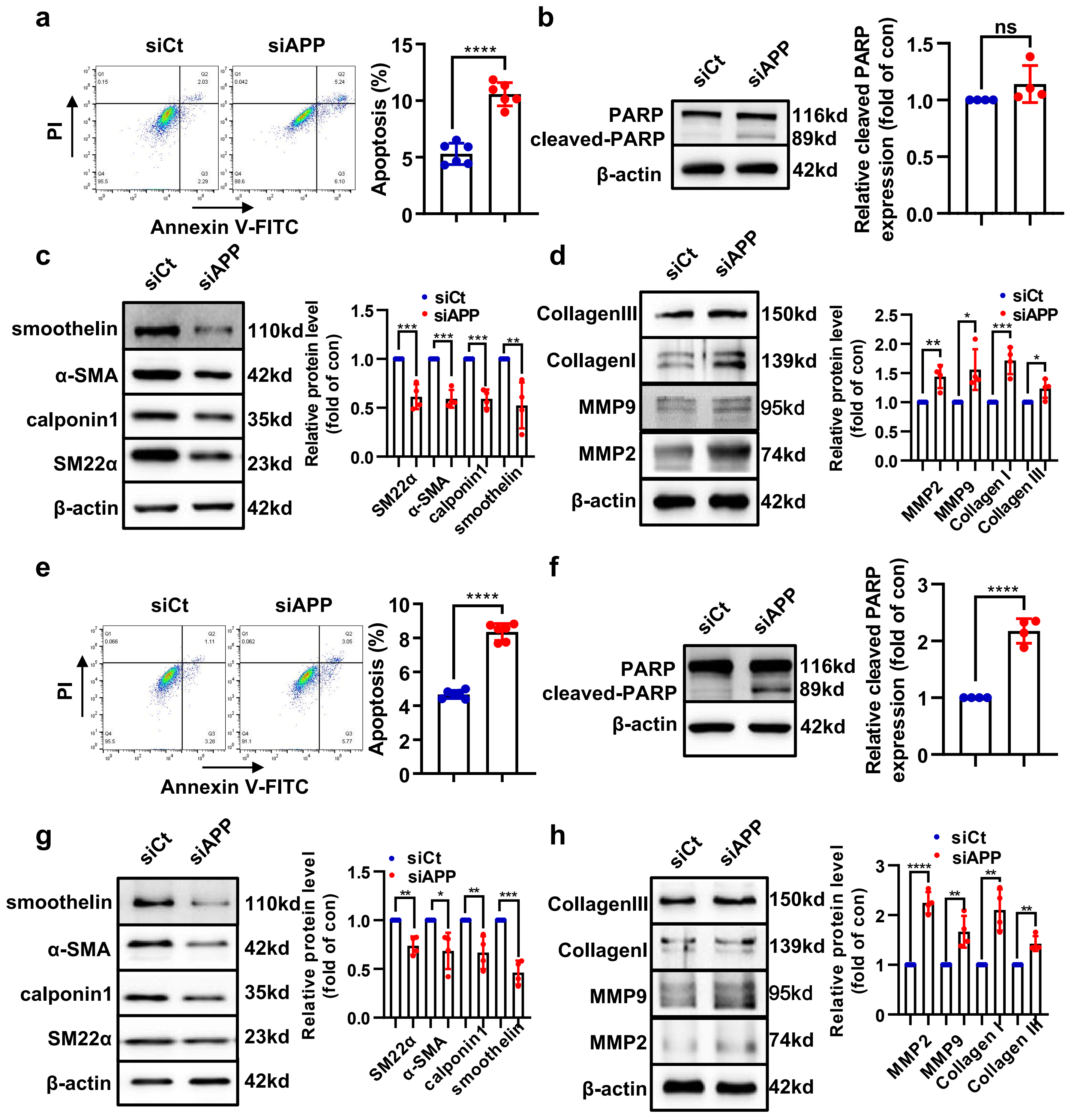

Supplement: Supplementary file 1 — Supplementary Figures [file 41440_2025_2315_MOESM1_ESM.pdf]
