# Supplementary material for: Copy number loss Of APP cause thoracic aortic dissection
Source: Hypertens Res. 2025 Aug 7;48(10):2641–53. doi: 10.1038/s41440-025-02315-8 (PMC12497644; doi:10.1038/s41440-025-02315-8)
Supplement: Supplementary file 2 — Supplementary Methods [file 41440_2025_2315_MOESM2_ESM.pdf]

Supplemental Methods

Whole-genome sequencing

DNA Sample testing: The quality of isolated genomic DNA was verified by using these three methods in combination: DNA purity and concentration were identified by NanoPhotometer® spectrophotometer (IMPLEN, CA, USA) (OD260/OD280). OD value of qualified sample is between 1.8-2.0. DNA degradation and suspected RNA/Protein contamination were verified by electrophoresis on 1% agarose gels**.** The concentration and purity of DNA samples were further quantified precisely by Qubit DNA Assay Kit in Qubit®2.0 Fluorometer (Life Technologies, CA, USA). A total amount of 1μg DNA per sample was required for library generation.

Library preparation and sequencing: A total amount of 1.0μg DNA per sample was used as input material for the DNA sample preparations. Sequencing libraries were generated using Truseq Nano DNA HT Sample Preparation Kit (Illumina USA) following manufacturer's recommendations and index codes were added to attribute sequences to each sample. The genomic DNA is randomly fragmented to a size of 350bp by Covaris cracker, then DNA fragments were end polished. A-tailed, and ligated with the full-length adapter for Illumina sequencing with further PCR amplification. At last, PCR products were purified (AMPure XP system) and libraries were analyzed for size distribution by Agilent2100 Bioanalyzer and quantified using real-time PCR. If library qualifies, the clustering of the index-coded samples was performed on a

cBot Cluster Generation System using Hiseq X HD PE Cluster Kit (Illumina) according to the manufacturer's instructions. After cluster generation, the library preparations were sequenced on an Illumina platform and paired-end reads were generated.

Quality Control: The raw image files obtained from Hiseq X were processed with Illumina pipeline for base calling and were stored as Fastq format (Raw data), which contain adapter contamination, low-quality nucleotide and undetected nucleotide (N). These sequence artifacts can impose significant influence on downstream processing analysis. Hence quality control, which is listed below, is applied to guarantee the meaningful downstream analysis. Filter reads with adapter contamination (>10 nucleotide aligned to the adapter, allowing ≤ 10% mismatches). Discard reads containing uncertain nucleotides more than 10 percentage. Discard the paired reads when single read has more than 50 percentage low quality (Phred quality <5) nucleotides. All the downstream bioinformatics analyses are based on high quality clean data, which can be obtained after these steps. At the same time, quality control statistics including total reads number, raw data, raw depth, sequencing error rate, percentage of reads with average quality> Q20, percentage of reads with average quality >Q30 and GC content distribution can be calculated.

Reads mapping to reference sequence: Valid sequencing data is mapped to the reference genome (UCSC hg19) by Burrows-Wheeler Aligner (BWA) software (Li H et al.2009-1) to get the original mapping result in BAM format. Subsequently, Samtools (Li H et al.2009-2) and Picard

(http://broadinstitute.github.io/picard) are respectively utilized to sort bam files, do duplicate-marking to generate final bam file. If one or one pair read(s) has multiple mapping positions, the strategy adopted by BWA is to select the best one, if there are multi best mapping position, we randomly pick one. Mapping step is very difficult due to mismatches, including true mutation and sequencing error, and duplicates resulted from PCR amplification. These duplicate reads are uninformative and shouldn't be considered as evidence for variants. Picard is employed to mark these duplicates so that we will ignore them in the following analysis.

Variant calling: In this step, reads that aligned to exon regions were collected for mutation identification and subsequent analysis. Samtools mpileup and bcftools are used to do variant calling and identify SNP, indels.

CNV refers to the increase or reduction of copy number of large fragments in the genome and is a very important molecular mechanism. There are two types of CNV: deletion and duplication. Since CNV detection are not so accurate, we employed the reliable user-friendly computational pipeline-Control-FREEC (Boeva V et al. 2012) to discover disruptive genic CNVs in human genetic studies of disease, which might be missed by standard approaches.

Functional Annotation: Functional annotation is very important because the link between genetic variation and disease can be found in this step. ANNOVAR (Wang K et al.2010) is performed to do annotation for VCF (Variant Call Format) file obtained in the previous step. The

variant position, variant type, conservative prediction and other information are obtained at this step through a variety of databases, such as dbSNP, 1000 Genome, ExAC, CADD and HGMD. Since we are interested in exonic variants, gene transcript annotation databases, such as Consensus CDS, RefSeq, Ensembl and UCSC, are also applied for annotation to determine amino acid alternation. CNV that we detected were annotated by CNVD (Copy Number Variation in Disease) and DGV (Database of Genomic Variants).

Filter: Variants obtained from previous steps are then filtered with the MAF>1% in the 1000 Genomes databases (1000 Genomes Project Consortium). Only SNVs occurring in exons or in canonical splice sites (splicing junction 10 bp) are further analyzed since we are interested in amino acid changes. Then synonymous SNVs which are not relevant to the amino acid alternation are discarded to get nonsynonymous SNVs, leading to different gene expression products. Finally, the retained nonsynonymous SNVs are submitted to PolyPhen-2 (Adzhubei I et al.2013), SIFT (Ng PC et al.2003), Mutation Taster (Schwarz J M et.al,2010), CADD (Martin K et.al,2014) for functional prediction, at least half of these four kinds of software show the SNV is not benign can it be retained.

CNV analysis and statistics

We used 100 cases and 132 controls as the discovery cohort for this association study project. Cases were recruited from patients with sporadic

TAD, control samples were confirmed without dissection. All the cases and controls were individuals of Chinese ancestry, we assessed the CNV in case and control in both two groups and summarized the statistics of CNV length and size in 100 cases samples. Single-tailed fisher's exact test was used for case-control analyses. The association P values were corrected by several multiple-testing correction methods including Bonferroni correction and Holm-Bonferroni method. False discovery rates were also calculated by Benjamini-Hochberg procedure and Benjamini-Yekutieli procedure. Odds ratio was calculated by CNV number count in case and control. Analyses were performed using statistical packages in R, Manhattan plot (according to Odds ratio value whether greater than 1 and QQ plot were performed by using the result of fisher's exact test.

MLPA and CNV Replication

Based on the whole genome sequencing results of discovery stage, we performed CNV association study and identified four candidate CNVs of type A aortic dissection. The four CNVs were further analyzed in 289 subjects (157 type A aortic dissections and 132 controls). According to the protocol of MLPA (MRC-Holland, Amsterdam, The Netherlands), we designed two or three pairs of probes matching highly conserved segments of target CNVs and reference genome (the probes and universal primers are listed in Supplementary table 4). The peak areas in

capillary electrophoresis of the three different products of each CNV were calculated to ensure accuracy of the experimental results. We used Chi-square test to estimate the CNV rate differences between cases and controls. The significance threshold was set at $P < 0.05$.

Human Thoracic Aortic Tissues

The human aortic tissue samples were collected in accordance with the guidelines approved by the Fuwai Hospital Ethics Committee. Informed consents were obtained for all the subject samples. The presence of aneurysm was confirmed at the time of surgery by experienced cardiothoracic surgeons, and the clinical phenotype diagnosis was confirmed by standard histopathology. Pathological human aortic samples were obtained from patients with aortic dissection undergoing surgical ascending aortic valve replacement or Bentall surgery and the control aortic tissue samples were obtained from donors without aortic disease.

Mice

The APP knockout ($APP^{-/-}$) mice were constructed on a C57BL/6 background using CRISPER/Cas9 technology, which were purchased from Cyagen Biosciences (Guangzhou) Inc. Briefly, the gRNA to mouse App gene, and Cas9 mRNA were co-injected into fertilized mouse eggs to

generate targeted knockout offspring. F0 founder animals were identified by PCR followed by sequence analysis, which were bred to wildtype mice to test germline transmission and F1 animal generation. The gRNA target sequences are listed as follow: gRNA1 (matching forward strand of gene): AATACTAGGTACAGCCGCTCAGG; gRNA2 (matching reverse strand of gene): GGAGGCCCTTTCATGCATTGTGG. The APP knockout mice were genotyped using the following primers: Forward primer (F1): 5'-TAAATGGCTCTCTTGAAGATGCAC-3'; Reverse primer (R1): 5'-GAGACCTCACATTATAGTCTTCCTG-3'. All animal experiments were confirmed to the guidelines and approved by the Institutional Animal Care and Use Committee (IACUC), National Center for Cardiovascular Diseases, Fuwai Hospital, Chinese Academy of Medical Sciences. All mice were housed in a sterile environment with 12 hours dark/light cycle and had free access to food and water.

Mouse TAD models

The PCSK9/ AngII model was constructed as previously described[43]. 10-12-week-old male mice were used in this study. The adeno-associated virus (AAV, serotype 8) containing PCSK9 D377Y was injected via tail vein at a dose of 3×10^{11} genome copy per mouse. After injection, the mice were fed with a western diet (HCD, 17.3% protein, 21.2% fat, 48.5% carbohydrate, 0.2% cholesterol by mass, and 42% calories from fat; TD.88137, Harlan) for 2 weeks. After 2 weeks, the mice were infused with AngII (1000ng/kg/min) via subcutaneously implanted mini-osmotic

pumps (Alzet, model 2004) for another 28 days to induce the development of TAD.

The BAPN model was performed as previously described[23]. Neutrophil-derived matrix metalloproteinase 9 triggers acute aortic dissection. Briefly, 3-week-old male mice were treated with 1g/kg/day of BAPN (Sigma-Aldrich) in drinking water for 28 days. The maximal diameter of the aorta was measured with a digital caliper in a double-blind way.

Cell culture

HASMCs were purchased from ScienCell and cultured in Smooth Muscle Cell Medium (ScienCell, USA) supplemented with 10% fetal bovine serum in 5% CO_2, 37°C, humidified atmosphere.

Isolation of primary MASMCs

Primary MASMCs were isolated from the thoracic aortas of $APP^{+/+}$ and $APP^{-/-}$ mice by collagenase digestion according to the previous method[44]. Briefly, thoracic aortas of three-week-old male mice were harvested from the aortic arch to the abdominal aorta. The intima and adventitia were stripped away gently. Then, the aortic tissues were minced and digested in serum-free Dulbecco's modified Eagle's medium (DMEM,

C11995500BT, Gibco) containing 1mg/ml type I collagenase (17018029, Gibco, MD, USA) for 4 hours at 37°C in a water bath shaken at 120 cycles/min. Subsequently, the suspensions were collected and centrifugated at 1000 rpm for 5 minutes, and then the isolated cells were cultured in DMEM supplemented with 10% fetal bovine serum (10091148, Gibco) and 100 U/ml penicillin-streptomycin and incubated in 5% CO_2, 37°C, humidified atmosphere.

Immunofluorescence Staining

Mouse aorta tissue samples were fixed with 4% paraformaldehyde and embedded in paraffin. Then, the samples were serially sectioned at 5 μm on a rotary microtome (RM2255, Leica) and collected on the glass slides. Heat mediated antigen retrieval using EDTA solution was applied on the sections. The sections were blocked in 5% goat serum with 0.3% Triton X-100 for 1 hour at room temperature and then incubated with primary antibodies (APP, 1:100, ab32136, Abcam; MMP2, 1:200, ab92536, Abcam; MMP9, ab38898, Abcam; collagen I, ab270993 and ab260043, Abcam) at 4℃ overnight. After washing with PBS for 3 times, the sections were incubated with Alexa-Fluor-594- and Alexa-Fluor-488-conjugated anti-rabbit or anti-mouse secondary antibodies (1:1000, Thermo Fisher Scientific) for 1 hour at room temperature. Then, the nuclei were stained with DAPI. The sections were imaged using confocal laser scanning microscope (TCS SP8, Leica).

H&E staining

The aortas of mice were dissected and fixed in 4% paraformaldehyde. The samples were dehydrated, embedded in paraffin, and sectioned at 5μm. Then the sections were dewaxed, hydrated and stained with H&E.

EVG staining

The aorta of mice was dissected and fixed in 4% paraformaldehyde, embedded in paraffin and sectioned at 5μm. The EVG stained for elastin was performed using Elastic Stain Kit (sigma, HT15-1KT) according to the manufacturer's protocol. Elastin degradation was graded as follow: 1, < 25% degradation; 2, 25% to 50% degradation; 3, 50% to 75% degradation; and 4, > 75% degradation or rupture[24].

Analysis of Cell Death by Annexin V/Propidium Iodide (PI) Staining

Apoptosis was measured by flow cytometry with the FITC Annexin V Apoptosis Detection Kit (556547, BD Biosciences) according to the manufacturer's protocol. Briefly, HASMCs and MASMCs were starved for 4 hours before drug treatment and subsequently they were treated

with AngII (1μmol/L) plus ox-LDL (150ng/mL) or BAPN (1 mmol/L) for 24 hours. After that, cells were digested with 0.25% trypsin (15050065, Life) and washed twice with sterilized PBS. Then, the collected cells were suspended with 1× binding buffer and stained with FITC Annexin V and Propidium Iodide (PI). Fluorescence was detected with the FACS Canto II Flow System, and all flow cytometry data were analyzed with FlowJo software (FlowJo LLC).

Terminal Deoxynucleotidyl Transferase dUTP Nick End Labeling (TUNEL) Assay Fixed sections of aorta were stained with In Situ Cell Death detection kit, POD (11684817910, Roche) according to the manufacture's protocol and counterstained with hematoxylin.

Quantitative reverse transcription-polymerase chain reaction (qRT-PCR)

Total RNAs from tissues and HASMCs were extracted with TRIzol reagent (15596018, Ambion, CA, USA). Reverse transcription was performed using the first-strand cDNA synthesis kit (Thermo Scientific, K1622). qRT-PCR was performed using a SYBR® Select Master Mix (Invitrogen) in a Vii7 Real-Time PCR System (Applied Biosystems, Foster City, MI, USA). The reaction condition was 95℃ for 10 minutes,

and 40 cycles of 95℃ for 15 seconds and 60℃ for 1 minute. GAPDH was used as the internal reference. The relative RNA levels were calculated with the $2^{-\Delta\Delta CT}$ method. The primer sequences used in this study were listed as follows:

APP-Forward, CCGAAACGAAAACCACCG and APP-Reverse, GGCATCAACAGGCTCAACT; GAPDH-Forward, GAAGGTGAAGGTCGGAGT and GAPDH-Reverse, CATGGGTGGAATCATATTGGAA.

Western blot analysis

The total protein of tissues and cells were lysed with RIPA lysis buffer (R0010, Solarbio, Beijing, China) supplemented with protease inhibitor (B14001, bimake) and phosphatase inhibitor (B15001, bimake). The concentrations of protein were measured by BCA Protein Assay Kit (23227, Thermo scientific, MA, USA). Proteins were separated by 8%-12% sodium dodecyl-polyacrylamide gel electrophoresis (SDS-PAGE) and transferred to nitrocellulose membranes (1620113, Bio-Rad, USA). Then, the membranes were blocked with 5% nonfat-dried milk in TBST (50 mM Tris-HCl, 150 mM NaCl and 0.1% Tween 20) for 1 hour at room temperature. Subsequently, the membranes were incubated with primary antibodies at 4℃ overnight. After washing with TBST for 3 times, membranes were incubated with horseradish peroxidase conjugated secondary antibodies (1:10000) for 1 hour at room temperature. Finally, chemiluminescence was performed with ECL reagent (Thermo Fisher).

siRNA-mediated gene knockdown

The siRNAs were obtained from RiboBio (Guangzhou, China). The siRNAs were listed as follows:

siRNA-negative control, sence, UUCUCCGAACGUGUCACGUTT and antisence, ACGUGACACGUUCGGAGAATT. siRNA-APP, sence, GGUCUUCAAUUACCAAGAATT and antisence, UUCUUGGUAAUUGAAGACCTT.

Cells were transfected with siRNAs with Lipofectamine 3000 (L3000015, Invitrogen, CA, USA) according to the manufacture's protocol.

RNA-seq

The primary MASMCs of $APP^{-/-}$ mice and $APP^{+/+}$ mice were treated with AngII (1μmol/L) plus ox-LDL (150 ng/mL) for 24 hours. Total RNA was isolated and purified using TRIzol reagent (Invitrogen, Carlsbad, CA, USA) following the manufacturer's procedure. The RNA amount and purity of each sample was quantified using NanoDrop ND-1000 (NanoDrop, Wilmington, DE, USA). The RNA integrity was assessed by Bioanalyzer 2100 (Agilent, CA, USA) with RIN number >7.0, and confirmed by electrophoresis with denaturing agarose gel. Poly (A) RNA is purified from 1μg total RNA using Dynabeads Oligo (dT)25-61005 (Thermo Fisher, CA, USA) using two rounds of purification. Then the

poly(A) RNA was fragmented into small pieces using Magnesium RNA Fragmentation Module (NEB, cat.e 6150, USA) under 94℃ 5-7min. Then the cleaved RNA fragments were reverse-transcribed to create the cDNA by SuperScript™ II Reverse Transcriptase (Invitrogen, cat. 1896649, USA), which were next used to synthesise U-labeled second-stranded DNAs with E. coli DNA polymerase I (NEB, cat.m0209, USA), RNase H (NEB, cat.m0297, USA) and dUTP Solution (Thermo Fisher, cat.R 0133, USA). An A-base is then added to the blunt ends of each strand, preparing them for ligation to the indexed adapters. Each adapter contains a T-base overhang for ligating the adapter to the A-tailed fragmented DNA. Single- or dual-index adapters are ligated to the fragments, and size selection was performed with AMPureXP beads. After the heat-labile UDG enzyme (NEB, cat.m0280, USA) treatment of the U-labeled second-stranded DNAs, the ligated products are amplified with PCR by the following conditions: initial denaturation at 95℃ for 3 minutes; 8 cycles of denaturation at 98℃ for 15 sec, annealing at 60℃ for 15 sec, and extension at 72℃ for 30 sec; and then final extension at 72℃ for 5 minutes. The average insert size for the final cDNA library was 300±50 bp. At last, we performed the 2×150bp paired-end sequencing (PE150) on an illumina Novaseq™ 6000 (LC-Bio Technology CO., Ltd., Hangzhou, China) following the vendor's recommended protocol.

Bioinformatics analysis of RNA-seq

Fastp software (https://github.com/OpenGene/fastp) were used to remove the reads that contained adaptor contamination, low quality bases and undetermined bases with default parameter. Then sequence quality was also verified using fastp. We used HISAT2 (https://ccb.jhu.edu/software/hisat2) to map reads to the reference genome of Mus musculus GRCm38(mm10). The mapped reads of each sample were assembled using StringTie (https://ccb.jhu.edu/software/stringtie) with default parameters. Then, all transcriptomes from all samples were merged to reconstruct a comprehensive transcriptome using gffcompare (https://github.com/gpertea/gffcompare/). After the final transcriptome was generated, StringTie and was used to estimate the expression levels of all transcripts. StringTie was used to perform expression level for mRNAs by calculating FPKM (FPKM = [total_exon_fragments/mapped_reads(millions) × exon_length(kB)]). The differentially expressed mRNAs were selected with fold change > 2 or fold change < 0.5 and with parametric F-test comparing nested linear models (p value < 0.05) by R package edgeR (https://bioconductor.org/packages/release/bioc/html/edgeR.html).

Materials and reagents

The material and reagents are as follows: Antibodies against APP (ab32136), β-actin(ab8226), MMP2 (ab92536), MMP9 (ab38898), Collagen I

(ab88147), Collagen III (ab184993, ab77778), SM22α (ab14106), calponin 1 (ab46794), α-SMA (ab7817, ab5694), Smoothelin (ab219652) were from Abcam (UK). The antibodies against GAPDH (#97166), cleaved caspase3(#9664S), PARP(#9542S), cleaved PARP (#5625S) were from Cell Signaling Technology (CST, Danvers, MA). BAPN (A3134) were from Sigma-Aldrich (St. Louis, MO). AngII (ab120183) were from Abcam (UK), Human ox-LDL (YB-002) was from Yiyuan (China), PCSK9 ELISA kit (ab215538) was from Abcam (UK). Dynabeads Protein G (10004D) was from Invitrogen (USA).

Supplementary figure legends

Supplementary Fig. 1. CNV distribution in type A aortic dissection individuals and CNV association study (OR<1).

(**a**), The number of CNVs located in different regions of human genome; cds, coding sequence; utr5, 5'-untranslated region; utr3, 3'-untranslated region. (**b**), The number of CNVs documented in common databases, including database of genomic variants (DGV), Copy Number Variation in Disease database (CNVD), Online Mendelian Inheritance in Man (CNVD), Genome Wide Association Study (GWAS), and Human Gene Mutation Database (HGMD). (**c**), CNVs categorized by different sizes. (**d**), A Manhattan plot of discovery stage genome-wide association results from comparison of TAD cases to healthy controls. For each tested CNV, the significance is displayed on the y-axis as the -log10 of the P

value. The -log10 results are ordered along the x-axis by chromosome, with each colored dot representing a different CNV. CNVs located in the same chromosome are presented with the same color. The association study suggested no CNV associated to low incidence of TAD (OR<1).

Supplementary Fig. 2. The expression of DSCAM, PROCR and LINC00907.

a, Expression of DSCAM and PROCR protein in the aorta of TAD patients and healthy individuals was determined using western blot. b, Expression of LINC00907 in the aorta of TAD patients and healthy individuals was determined using RT-PCR. Data were presented as Mean±S.E.M.

Supplemental Fig. 3. The generation of APP knockout mice and elastin degradation grades.

(**a**), APP knockout ($APP^{-/-}$) C57BL/6 mice were constructed based on CRISPR/Cas9 technology. (**b**), Expression of APP in aorta of WT mice and $APP^{-/-}$ mice was determined by western blot. (**c**), Elastin degradation grades were determined by EVG staining: Grade 1, < 25% degradation; Grade 2, 25% to 50% degradation; Grade 3, 50% to 75% degradation; and Grade 4, > 75% degradation, dissection or rupture. Data were presented as Mean±S.E.M. ****P<0.0001.

Supplemental Fig. 4. Knockdown efficiency of siRNA

HASMCs were transfected with siRNA-negative control (siCt) or siRNA-APP (siAPP). After 48 hours, the cells were harvested and knockdown efficiency of siRNA was measured by real time quantitative polymerase chain reaction (qRT-PCR). Data were presented as Mean±S.E.M. ****P<0.0001.

Supplemental Fig. 5. APP knockout or knockdown cannot affect apoptosis and secretory phenotypic of SMCs without external stimuli

Primary MASMCs were isolated from the $APP^{-/-}$ mice and WT littermates. HASMCs were transfected with siCt or siAPP for 48 hours. (**a**), Expression of cleaved PARP in MASMCs was examined using western blot. (**b**), Expression of cleaved PARP in HASMCs was examined using western blot. (**c**), Expression of contractile markers including SM22α, calponin-1, α-SMA and smoothelin in MASMCs were examined using western blot. (**d**), Expression of secretory markers including MMP2, MMP9, Collagen I and Collagen III in MASMCs were examined using western blot. (**e**), Expression of contractile markers including SM22α, calponin-1, α-SMA and smoothelin in HASMCs treated with BAPN were examined using western blot. (**f**), Expression of secretory markers including MMP2, MMP9, Collagen I and Collagen III in HASMCs treated

with BAPN were examined using western blot. Data were presented as Mean±S.E.M. HASMCs, Human aortic smooth muscle cells. siCt, siRNA-negative control. PARP, poly (ADP-ribose) polymerase.

Supplemental Fig. 6. APP knockdown promoted apoptosis and secretory phenotypic of HASMCs

HASMCs were transfected with siCt or siAPP. After 48 hours, the cells were treated with BAPN (1 mmol/L) or AngII (1 μmol/L) plus ox-LDL (150 ng/ml) for 24 hours. (**a**), Cell apoptosis in HASMCs treated with BAPN was examined using annexin V/ PI staining and flow cytometry analysis. (**b**), Expression of cleaved PARP in HASMCs treatment with BAPN was examined using western blot. (**c**), Expression of contractile markers including SM22α, calponin-1, α-SMA and smoothelin in HASMCs treated with BAPN were examined using western blot. (**d**), Expression of secretory markers including MMP2, MMP9, Collagen I and Collagen III in HASMCs treated with BAPN were examined using western blot. (**e and f**), Cell apoptosis in HASMCs treatment with AngII plus ox-LDL was examined using Annexin V/ PI staining and flow cytometry analysis (**e**) and western blot (**f**). (**g and h**), Expression of contractile markers (**g**) and secretory markers (**h**) in HASMCs treated with AngII plus ox-LDL were examined using western blot. Data were presented as Mean±S.E.M. *P<0.05, **P<0.01, ***P<0.001, ****P<0.0001. Scale bar = 100 μm. HASMCs, Human aortic smooth muscle cells. siCt, siRNA-negative control. Ox-LDL, oxidized low-density lipoprotein. PI,

propidium iodide, PARP, poly (ADP-ribose) polymerase.

Supplementary tables

Supplementary Table 1. Total CNVs identified by CNV burden analysis.

CHR	Start	End	CNV Type	Gene	Func	cytoBand	Con_shared_No.	Cas_shared_No.	P	OR	BONF	HOLM	FDR_BH
21	30213500	30214000	gain	.	intergenic	21q21.3	4	20	0.000658108	5.580992	0.279037962	0.279037962	0.158484275

21	41702000	41702500	gain	DSCAM	intronic	21q22.2	1	13	0.00093892	13.75934	0.398102165	0.397163245	0.158484275
17	30669500	30671500	gain	.	upstream	17q11.2	10	29	0.001121351	3.409734	0.475452824	0.473210122	0.158484275
18	26594000	26788500	loss	.	intergenic	18q12.1	1	11	0.003425618	11.38922	1	1	0.363115455
21	27379500	27380500	loss	APP	intronic	21q21.3	0	8	0.004328428	Inf	1	1	0.367050652

11	24543500	24561000	loss	LUZP2	intronic	11p14.3	0	7	0.00 8703 397	Inf	1	1	0.41 0026 717
11	82048000	82092000	loss	.	intergenic	11q14.1	0	7	0.00 8703 397	Inf	1	1	0.41 0026 717
18	58466500	58514000	loss	.	intergenic	18q21.3 2	0	7	0.00 8703 397	Inf	1	1	0.41 0026 717
5	50803500	50865500	loss	.	intergenic	5q11.2	0	7	0.00 8703 397	Inf	1	1	0.41 0026 717

18	40153000	40174500	loss	LINC00907	ncRNA_exonic	18q12.3	0	6	0.017406795	Inf	1	1	0.708891947
13	28505000	28514000	loss	.	intergenic	13q12.2	2	10	0.021470769	5.074155	1	1	0.708891947
21	17363000	17364500	gain	.	intergenic	21q21.1	1	8	0.021828307	8.020131	1	1	0.708891947
13	69877000	69896500	loss	.	intergenic	13q21.33	0	5	0.03463036	Inf	1	1	0.708891947

13	72989000	73014000	loss	.	intergenic	13q21.33	0	5	0.03463036	Inf	1	1	0.708891947
19	57532000	57533000	gain	.	intergenic	19q13.43	0	5	0.03463036	Inf	1	1	0.708891947
20	33754500	33764500	gain	PROCR	exonic	20q11.22	0	5	0.03463036	Inf	1	1	0.708891947
18	38644000	38801500	loss	.	intergenic	18q12.3	1	7	0.039328186	6.944812	1	1	0.708891947

11	95357500	95368000	loss	.	intergenic	11q21	2	8	0.06 1415 871	3.97 E+0 0	1	1	0.70 8891 947
5	152075000	152079500	loss	AC091969. 1	ncRNA_int ronic	5q33.1	2	8	0.06 1415 871	3.97 E+0 0	1	1	0.70 8891 947
12	91399500	91480500	loss	KERA	exonic	12q21.3 3	0	4	0.06 8539 254	Inf	1	1	0.70 8891 947
13	38429000	38441500	loss	TRPC4	intronic	13q13.3	0	4	0.06 8539 254	Inf	1	1	0.70 8891 947

13	59129500	59145000	loss	.	intergenic	13q21.1	0	4	0.06 8539 254	Inf	1	1	0.70 8891 947
13	79885500	79900000	loss	RBM26	exonic	13q31.1	0	4	0.06 8539 254	Inf	1	1	0.70 8891 947
14	42445000	42474000	loss	.	intergenic	14q21.1	0	4	0.06 8539 254	Inf	1	1	0.70 8891 947
15	46723000	46780500	loss	.	intergenic	15q21.1	0	4	0.06 8539 254	Inf	1	1	0.70 8891 947

2	68741500	68753000	loss	APLF	intronic	2p13.3	0	4	0.06 8539 254	Inf	1	1	0.70 8891 947
21	15821000	15847500	loss	.	intergenic	21q11.2	0	4	0.06 8539 254	Inf	1	1	0.70 8891 947
3	82962000	83224500	loss	.	intergenic	3p12.2	0	4	0.06 8539 254	Inf	1	1	0.70 8891 947
8	113891500	113928500	loss	CSMD3	intronic	8q23.3	0	4	0.06 8539 254	Inf	1	1	0.70 8891 947

18	71134000	71175000	loss	.	intergenic	18q22.3	1	5	0.12<br>0748<br>185	4.86<br>E+0<br>0	1	1	0.70<br>8891<br>947
9	79184500	79188000	gain	.	intergenic	9q21.13	1	5	0.12<br>0748<br>185	4.86<br>E+0<br>0	1	1	0.70<br>8891<br>947
10	69484000	69490500	loss	.	intergenic	10q21.3	28	38	0.14<br>5682<br>869	1.44<br>E+0<br>0	1	1	0.70<br>8891<br>947
1	107230000	107287500	loss	.	intergenic	1p13.3	0	3	0.13<br>4958<br>736	Inf	1	1	0.70<br>8891<br>947

14	27043000	27062500	loss	NOVA1	intronic	14q12	0	3	0.134958736	Inf	1	1	0.708891947
15	46949000	46959500	loss	.	intergenic	15q21.1	0	3	0.134958736	Inf	1	1	0.708891947
16	64611000	64644000	loss	.	intergenic	16q21	0	3	0.134958736	Inf	1	1	0.708891947
18	41193500	41204500	loss	.	intergenic	18q12.3	0	3	0.134958736	Inf	1	1	0.708891947

2	166852500	166875000	loss	SCN1A	exonic	2q24.3	0	3	0.134958736	Inf	1	1	0.708891947
21	22600000	22607500	loss	NCAM2	intronic	21q21.1	0	3	0.134958736	Inf	1	1	0.708891947
3	35365000	35399000	loss	.	intergenic	3p22.3	0	3	0.134958736	Inf	1	1	0.708891947
5	124949000	124956000	loss	.	intergenic	5q23.2	3	7	0.191955509	2.27E+00	1	1	0.708891947

20	42760000	42764500	loss	JPH2	intronic	20q13.12	7	12	0.205323006	1.69E+00	1	1	0.708891947
5	89201000	89272500	loss	.	intergenic	5q14.3	1	4	0.204174829	3.85E+00	1	1	0.708891947
6	126751500	127073000	loss	CENPW	intergenic	6q22.32	1	4	0.204174829	3.85E+00	1	1	0.708891947
19	44807000	44807500	gain	ZNF235	intronic	19q13.31	2	5	0.248550703	2.41E+00	1	1	0.708891947

1	83176000	83238500	loss	.	intergenic	1p31.1	0	2	0.264408953	Inf	1	1	0.708891947
10	62840000	62914000	loss	.	intergenic	10q21.2	0	2	0.264408953	Inf	1	1	0.708891947
11	134828500	135006516	gain	.	intergenic	11q25	0	2	0.264408953	Inf	1	1	0.708891947
11	26760000	26785000	loss	.	intergenic	11p14.2	0	2	0.264408953	Inf	1	1	0.708891947

11	71796000	71828000	gain	LAMTOR1,LRTOMT,ANAPC15	exonic	11q13.4	0	2	0.264408953	Inf	1	1	0.708891947
12	24082500	24095000	loss	SOX5	intronic	12p12.1	0	2	0.264408953	Inf	1	1	0.708891947
12	29088000	29117000	loss	.	intergenic	12p11.22	0	2	0.264408953	Inf	1	1	0.708891947
13	35933500	35945000	loss	NBEA	intronic	13q13.3	0	2	0.264408953	Inf	1	1	0.708891947

13	61825000	61840000	loss	.	intergenic	13q21.2	0	2	0.26 4408 953	Inf	1	1	0.70 8891 947
13	93374000	93387000	loss	GPC5	intronic	13q31.3	0	2	0.26 4408 953	Inf	1	1	0.70 8891 947
15	49174500	49329000	gain	SECISBP2 L,SHC4	exonic	15q21.1	0	2	0.26 4408 953	Inf	1	1	0.70 8891 947
15	63758000	63845000	loss	USP3	exonic	15q22.3 1	0	2	0.26 4408 953	Inf	1	1	0.70 8891 947

17	34102000	34119500	loss	MMP28	exonic	17q12	0	2	0.26 4408 953	Inf	1	1	0.70 8891 947
17	35426500	35437000	loss	.	intergenic	17q12	0	2	0.26 4408 953	Inf	1	1	0.70 8891 947
18	32495500	32496500	gain	.	intergenic	18q12.1	0	2	0.26 4408 953	Inf	1	1	0.70 8891 947
18	36744000	36774000	loss	.	intergenic	18q12.2	0	2	0.26 4408 953	Inf	1	1	0.70 8891 947

18	63068500	63090000	loss	.	intergenic	18q22.1	0	2	0.26 4408 953	Inf	1	1	0.70 8891 947
19	30996000	30996500	gain	ZNF536	intronic	19q12	0	2	0.26 4408 953	Inf	1	1	0.70 8891 947
19	51562500	51563000	gain	KLK13	intronic	19q13.4 1	0	2	0.26 4408 953	Inf	1	1	0.70 8891 947
2	24066500	24229000	loss	UBXN2A, ATAD2B	exonic	2p23.3	0	2	0.26 4408 953	Inf	1	1	0.70 8891 947

2	81653500	81690500	loss	AC012075.2	ncRNA_exonic	2p12	0	2	0.264408953	Inf	1	1	0.708891947
20	13858000	13879500	gain	SEL1L2	exonic	20p12.1	0	2	0.264408953	Inf	1	1	0.708891947
20	23333500	23346500	gain	GZF1,NXT1,RP3-322G13.5	exonic	20p11.21	0	2	0.264408953	Inf	1	1	0.708891947
21	26002500	26003000	gain	.	intergenic	21q21.2	0	2	0.264408953	Inf	1	1	0.708891947

21	36221500	36222000	gain	RUNX1	intronic	21q22.12	0	2	0.264408953	Inf	1	1	0.708891947
3	161709500	161758500	loss	.	intergenic	3q26.1	0	2	0.264408953	Inf	1	1	0.708891947
5	90896000	90936500	loss	.	intergenic	5q14.3	0	2	0.264408953	Inf	1	1	0.708891947
6	151507000	151519000	loss	.	intergenic	6q25.1	0	2	0.264408953	Inf	1	1	0.708891947

7	126072000	126157000	loss	GRM8	exonic	7q31.33	0	2	0.264408953	Inf	1	1	0.708891947
8	116806000	116825500	loss	.	intergenic	8q23.3	0	2	0.264408953	Inf	1	1	0.708891947
9	15838500	15958500	loss	CCDC171	exonic	9p22.3	0	2	0.264408953	Inf	1	1	0.708891947
4	165939500	166045500	loss	TMEM192, TRIM60	exonic	4q32.3	4	7	0.304936146	1.69E+00	1	1	0.708891947

18	50951000	50959500	loss	DCC	intronic	18q21.2	5	8	0.324702015	1.54E+00	1	1	0.708891947
10	105052500	105147000	loss	TAF5,PCGF6	exonic	10q24.33	1	3	0.334217184	2.86E+00	1	1	0.708891947
13	53924500	53968500	loss	.	intergenic	13q14.3	1	3	0.334217184	2.86E+00	1	1	0.708891947
13	83021500	83139000	loss	.	intergenic	13q31.1	1	3	0.334217184	2.86E+00	1	1	0.708891947

15	46267500	46344000	loss	.	intergenic	15q21.1	1	3	0.334217184	2.86E+00	1	1	0.708891947
21	34280500	34281000	gain	.	intergenic	21q22.11	1	3	0.334217184	2.86E+00	1	1	0.708891947
7	2353000	2393000	loss	SNX8	exonic	7p22.3	1	3	0.334217184	2.86E+00	1	1	0.708891947
9	88821500	88844000	loss	C9orf153	exonic	9q21.33	1	3	0.334217184	2.86E+00	1	1	0.708891947

22	39009000	39009500	gain	FAM227A	intronic	22q13.1	7	9	0.448676586	1.23E+00	1	1	0.708891947
1	157803000	157854500	loss	CD5L	exonic	1q23.1	0	1	0.515463918	Inf	1	1	0.708891947
1	173260500	173285500	loss	LOC100506023	ncRNA_intronic	1q25.1	0	1	0.515463918	Inf	1	1	0.708891947
1	190563000	190639000	loss	RP11-463J7.2	ncRNA_exonic	1q31.1	0	1	0.515463918	Inf	1	1	0.708891947

1	227688000	227784000	gain	ZNF678	exonic	1q42.13	0	1	0.51 5463 918	Inf	1	1	0.70 8891 947
1	69883000	69914500	loss	.	intergenic	1p31.1	0	1	0.51 5463 918	Inf	1	1	0.70 8891 947
1	75231000	75320000	loss	TYW3	UTR3	1p31.1	0	1	0.51 5463 918	Inf	1	1	0.70 8891 947
1	90827000	91029500	loss	.	intergenic	1p22.2	0	1	0.51 5463 918	Inf	1	1	0.70 8891 947

1	96039500	96069000	loss	.	intergenic	1p21.3	0	1	0.51 5463 918	Inf	1	1	0.70 8891 947
10	11847000	11901500	gain	PROSER2, PROSER2-AS1	exonic	10p14	0	1	0.51 5463 918	Inf	1	1	0.70 8891 947
10	129860500	129867000	gain	PTPRE	exonic	10q26.2	0	1	0.51 5463 918	Inf	1	1	0.70 8891 947
10	4360000	4371000	loss	.	intergenic	10p15.1	0	1	0.51 5463 918	Inf	1	1	0.70 8891 947

10	44258000	44269000	loss	.	intergenic	10q11.21	0	1	0.515463918	Inf	1	1	0.708891947
10	61613000	61631500	loss	CCDC6	intronic	10q21.2	0	1	0.515463918	Inf	1	1	0.708891947
10	62641500	62716000	gain	RHOBTB1	exonic	10q21.2	0	1	0.515463918	Inf	1	1	0.708891947
10	82936000	82951000	loss	.	intergenic	10q23.1	0	1	0.515463918	Inf	1	1	0.708891947

10	84042500	84062500	loss	NRG3	intronic	10q23.1	0	1	0.515463918	Inf	1	1	0.708891947
10	8800500	8818500	loss	.	intergenic	10p14	0	1	0.515463918	Inf	1	1	0.708891947
10	98561000	98561500	gain	.	intergenic	10q24.1	0	1	0.515463918	Inf	1	1	0.708891947
11	10511000	10538000	gain	MTRNR2L8,RNF141,AMPD3,M	exonic	11p15.4	0	1	0.515463918	Inf	1	1	0.708891947

				IR4485									
11	106714500	106724000	loss	GUCY1A2	intronic	11q22.3	0	1	0.51 5463 918	Inf	1	1	0.70 8891 947
11	109335000	109349500	loss	.	intergenic	11q22.3	0	1	0.51 5463 918	Inf	1	1	0.70 8891 947
11	109428000	109436000	loss	.	intergenic	11q22.3	0	1	0.51 5463 918	Inf	1	1	0.70 8891 947
11	119072500	119165500	loss	CBL	exonic	11q23.3	0	1	0.51 5463	Inf	1	1	0.70 8891

									918				947
11	12116500	12872500	gain	TEAD1,PARVA,MIR6124,MICALCL,MICAL2	exonic	11p15.3-p15.2	0	1	0.515463918	Inf	1	1	0.708891947
11	121834500	121912000	loss	.	intergenic	11q24.1	0	1	0.515463918	Inf	1	1	0.708891947
11	122334000	122358000	loss	.	intergenic	11q24.1	0	1	0.515463918	Inf	1	1	0.708891947

11	123577500	123595500	gain	ZNF202	UTR3	11q24.1	0	1	0.51 5463 918	Inf	1	1	0.70 8891 947
11	126345500	127032000	gain	KIRREL3, MIR3167, KIRREL3-AS2,KIRREL3-AS3	exonic	11q24.2	0	1	0.51 5463 918	Inf	1	1	0.70 8891 947
11	14786500	14794500	loss	PDE3B	exonic	11p15.2	0	1	0.51 5463 918	Inf	1	1	0.70 8891 947
11	19322000	20139500	gain	NAV2-AS	exonic	11p15.1	0	1	0.51		1	1	0.70

				4,NAV2-AS5,NAV2,MIR4486,LOC100126784,MIR4694					5463918	Inf			8891947
11	28225500	28244500	loss	METTL15	exonic	11p14.1	0	1	0.515463918	Inf	1	1	0.708891947
11	3878500	3943500	gain	STIM1	intronic	11p15.4	0	1	0.515463918	Inf	1	1	0.708891947

11	42016000	42026500	loss	.	intergenic	11p12	0	1	0.515463918	Inf	1	1	0.708891947
11	43647000	43649500	gain	.	intergenic	11p11.2	0	1	0.515463918	Inf	1	1	0.708891947
11	59161000	59188000	loss	.	intergenic	11q12.1	0	1	0.515463918	Inf	1	1	0.708891947
11	79980500	80051500	loss	.	intergenic	11q14.1	0	1	0.515463918	Inf	1	1	0.708891947

11	84151500	84184500	loss	DLG2	intronic	11q14.1	0	1	0.515463918	Inf	1	1	0.708891947
11	96534000	96545500	loss	.	intergenic	11q21	0	1	0.515463918	Inf	1	1	0.708891947
11	98398500	98401000	gain	.	intergenic	11q22.1	0	1	0.515463918	Inf	1	1	0.708891947
12	100186000	100215500	loss	ANKS1B	exonic	12q23.1	0	1	0.515463918	Inf	1	1	0.708891947

12	101205500	101234000	loss	ANO4	intronic	12q23.1	0	1	0.515463918	Inf	1	1	0.708891947
12	15142500	15185500	loss	.	intergenic	12p12.3	0	1	0.515463918	Inf	1	1	0.708891947
12	19908000	19940000	gain	.	intergenic	12p12.3	0	1	0.515463918	Inf	1	1	0.708891947
12	47159000	47214500	loss	SLC38A4	exonic	12q13.11	0	1	0.515463918	Inf	1	1	0.708891947

12	55389500	55408500	gain	.	intergenic	12q13.2	0	1	0.515463918	Inf	1	1	0.708891947
12	55481500	55490000	loss	.	intergenic	12q13.2	0	1	0.515463918	Inf	1	1	0.708891947
12	89317500	89348000	loss	.	intergenic	12q21.33	0	1	0.515463918	Inf	1	1	0.708891947
12	90809000	90867000	loss	.	intergenic	12q21.33	0	1	0.515463918	Inf	1	1	0.708891947

13	102483000	102519500	loss	FGF14	intronic	13q33.1	0	1	0.51 5463 918	Inf	1	1	0.70 8891 947
13	38929000	38946000	loss	UFM1	exonic	13q13.3	0	1	0.51 5463 918	Inf	1	1	0.70 8891 947
13	39142000	39360000	gain	FREM2,LI NC00366	exonic	13q13.3	0	2	0.26 4408 953	Inf	1	1	0.70 8891 947
13	41968000	41984000	gain	.	intergenic	13q14.1 1	0	1	0.51 5463 918	Inf	1	1	0.70 8891 947

13	44714000	44724500	gain	SMIM2-IT1,SMIM2,SMIM2-AS1	exonic	13q14.11	0	1	0.515463918	Inf	1	1	0.708891947
13	70893000	70943500	loss	.	intergenic	13q21.33	0	1	0.515463918	Inf	1	1	0.708891947
13	72572500	72573000	gain	.	intergenic	13q21.33	0	1	0.515463918	Inf	1	1	0.708891947
13	72855500	72889500	loss	.	intergenic	13q21.33	0	1	0.515463918	Inf	1	1	0.708891947

13	73183500	73198000	loss	.	intergenic	13q21.33	0	1	0.515463918	Inf	1	1	0.708891947
13	80318000	80391000	gain	.	intergenic	13q31.1	0	1	0.515463918	Inf	1	1	0.708891947
13	85333000	85342500	loss	.	intergenic	13q31.1	0	1	0.515463918	Inf	1	1	0.708891947
13	86383500	86391500	loss	.	intergenic	13q31.1	0	1	0.515463918	Inf	1	1	0.708891947

13	88332500	88342000	loss	.	downstream	13q31.2	0	1	0.515463918	Inf	1	1	0.708891947
13	88777000	88797000	gain	.	intergenic	13q31.2	0	1	0.515463918	Inf	1	1	0.708891947
14	104671500	104814000	gain	.	intergenic	14q32.33	0	1	0.515463918	Inf	1	1	0.708891947
14	23788500	23793500	gain	PABPN1,BCL2L2-PABPN1	exonic	14q11.2	0	1	0.515463918	Inf	1	1	0.708891947

14	35869500	35876500	gain	NFKBIA	exonic	14q13.2	0	1	0.515463918	Inf	1	1	0.708891947
14	37769000	37776500	loss	MIPOL1	intronic	14q13.3	0	1	0.515463918	Inf	1	1	0.708891947
14	68771000	68786500	gain	RAD51B	intronic	14q24.1	0	1	0.515463918	Inf	1	1	0.708891947
14	80388000	80644000	loss	.	intergenic	14q31.1	0	1	0.515463918	Inf	1	1	0.708891947

14	98907000	98946500	gain	.	intergenic	14q32.2	0	1	0.515463918	Inf	1	1	0.708891947
15	45910500	45911500	gain	.	intergenic	15q21.1	0	1	0.515463918	Inf	1	1	0.708891947
15	47429500	47444500	loss	.	intergenic	15q21.1	0	1	0.515463918	Inf	1	1	0.708891947
15	48483000	48505000	loss	CTXN2,SLC12A1	exonic	15q21.1	0	1	0.515463918	Inf	1	1	0.708891947

15	56962500	56970500	loss	ZNF280D	exonic	15q21.3	0	1	0.515463918	Inf	1	1	0.708891947
15	69870500	69887500	loss	.	intergenic	15q23	0	1	0.515463918	Inf	1	1	0.708891947
15	81073500	81245500	gain	CEMIP,MIR549	exonic	15q25.1	0	1	0.515463918	Inf	1	1	0.708891947
15	83897500	83901000	loss	.	intergenic	15q25.2	0	1	0.515463918	Inf	1	1	0.708891947

15	88013000	88043000	loss	.	intergenic	15q25.3	0	1	0.51<br>5463<br>918	Inf	1	1	0.70<br>8891<br>947
15	88461500	88938000	gain	NTRK3,N<br>TRK3-AS1	exonic	15q25.3	0	1	0.51<br>5463<br>918	Inf	1	1	0.70<br>8891<br>947
15	95656000	95688500	loss	.	intergenic	15q26.2	0	1	0.51<br>5463<br>918	Inf	1	1	0.70<br>8891<br>947
16	11103000	11106000	gain	CLEC16A	intronic	16p13.1<br>3	0	1	0.51<br>5463<br>918	Inf	1	1	0.70<br>8891<br>947

16	26072500	26112500	gain	HS3ST4	intronic	16p12.1	0	1	0.515463918	Inf	1	1	0.708891947
16	48952000	48964500	gain	.	intergenic	16q12.1	0	1	0.515463918	Inf	1	1	0.708891947
16	52995000	52996000	gain	.	intergenic	16q12.2	0	1	0.515463918	Inf	1	1	0.708891947
16	64039000	64074500	loss	.	intergenic	16q21	0	1	0.515463918	Inf	1	1	0.708891947

16	64808500	64830500	loss	.	intergenic	16q21	0	1	0.515463918	Inf	1	1	0.708891947
16	80940000	80954500	loss	.	intergenic	16q23.2	0	1	0.515463918	Inf	1	1	0.708891947
16	81749500	81765000	loss	.	intergenic	16q23.3	0	1	0.515463918	Inf	1	1	0.708891947
17	11601000	11602000	gain	DNAH9	intronic	17p12	0	1	0.515463918	Inf	1	1	0.708891947

17	31235500	32341500	gain	ASIC2,AA06,SPACA3,TMEM98	exonic	17q12-q11.2	0	1	0.515463918	Inf	1	1	0.708891947
17	33478000	33478500	gain	UNC45B	intronic	17q12	0	1	0.515463918	Inf	1	1	0.708891947
17	43300000	43308500	gain	FMNL1	exonic	17q21.31	0	1	0.515463918	Inf	1	1	0.708891947
17	56589000	56599500	gain	MTMR4,SEPT4	exonic	17q22	0	1	0.515463918	Inf	1	1	0.708891947

17	69440000	69455500	loss	.	intergenic	17q24.3	0	1	0.515463918	Inf	1	1	0.708891947
18	20502500	20525500	loss	MIR4741, RBBP8	exonic	18q11.2	0	1	0.515463918	Inf	1	1	0.708891947
18	30674000	30702000	loss	CCDC178	intronic	18q12.1	0	1	0.515463918	Inf	1	1	0.708891947
18	32565500	32568500	gain	MAPRE2	intronic	18q12.1	0	1	0.515463918	Inf	1	1	0.708891947

18	32594000	32602000	loss	MAPRE2	intronic	18q12.1	0	1	0.515463918	Inf	1	1	0.708891947
18	36366000	36417500	loss	.	intergenic	18q12.2	0	1	0.515463918	Inf	1	1	0.708891947
18	36596500	36619000	loss	.	intergenic	18q12.2	0	1	0.515463918	Inf	1	1	0.708891947
18	39084500	39127000	loss	KC6	ncRNA_exonic	18q12.3	0	1	0.515463918	Inf	1	1	0.708891947

18	41760500	41772000	loss	.	intergenic	18q12.3	0	1	0.515463918	Inf	1	1	0.708891947
18	59487500	59512500	gain	RNF152	intronic	18q21.33	0	1	0.515463918	Inf	1	1	0.708891947
18	61023500	61028500	loss	KDSR	exonic	18q21.33	0	1	0.515463918	Inf	1	1	0.708891947
18	62609500	62638500	loss	.	intergenic	18q22.1	0	1	0.515463918	Inf	1	1	0.708891947

18	62681500	62691000	gain	.	intergenic	18q22.1	0	1	0.51 5463 918	Inf	1	1	0.70 8891 947
18	62772500	62784000	loss	.	intergenic	18q22.1	0	1	0.51 5463 918	Inf	1	1	0.70 8891 947
2	157739500	157769000	loss	.	intergenic	2q24.1	0	1	0.51 5463 918	Inf	1	1	0.70 8891 947
2	178602500	178700500	gain	PDE11A	exonic	2q31.2	0	1	0.51 5463 918	Inf	1	1	0.70 8891 947

2	193186500	193207500	loss	.	intergenic	2q32.3	0	1	0.51 5463 918	Inf	1	1	0.70 8891 947
2	220070500	220121000	gain	GLB1L,ABCB6,ZFAND2B,ANKZF1,TUBA4A,ATG9A,TUBA4B,STK16	exonic	2q35	0	1	0.51 5463 918	Inf	1	1	0.70 8891 947
2	52038500	52200500	loss	.	intergenic	2p16.3	0	1	0.51 5463	Inf	1	1	0.70 8891

									918				947
2	85130000	85261500	loss	TMSB10,KCMF1	exonic	2p11.2	0	1	0.515463918	Inf	1	1	0.708891947
20	1323500	1338000	loss	SDCBP2-AS1,FKBP1A-SDCBP2	ncRNA_intronic	20p13	0	1	0.515463918	Inf	1	1	0.708891947
20	19422500	19769000	gain	SLC24A3	exonic	20p11.23	0	1	0.515463918	Inf	1	1	0.708891947
20	22163500	22172500	loss	.	intergenic	20p11.22	0	1	0.515463	Inf	1	1	0.708891

									918				947
20	51653000	51653500	gain	TSHZ2	intronic	20q13.2	0	1	0.515463918	Inf	1	1	0.708891947
20	58081000	58087500	loss	.	intergenic	20q13.32	0	1	0.515463918	Inf	1	1	0.708891947
20	59363000	59381500	gain	.	intergenic	20q13.33	0	1	0.515463918	Inf	1	1	0.708891947
20	59958500	59959000	gain	CDH4	intronic	20q13.33	0	1	0.515463	Inf	1	1	0.708891

									918				947
20	60103000	60126500	loss	CDH4	intronic	20q13.3 3	0	1	0.51 5463 918	Inf	1	1	0.70 8891 947
20	6024500	6027000	loss	LRRN4	exonic	20p12.3	0	1	0.51 5463 918	Inf	1	1	0.70 8891 947
21	17125500	17179500	loss	USP25	exonic	21q21.1	0	1	0.51 5463 918	Inf	1	1	0.70 8891 947
21	23427500	23458500	loss	AP000472. 2	ncRNA_int ronic	21q21.1	0	1	0.51 5463	Inf	1	1	0.70 8891

									918				947
21	25978500	25992000	loss	.	intergenic	21q21.2	0	1	0.515463918	Inf	1	1	0.708891947
21	26093000	26105500	loss	.	intergenic	21q21.2	0	1	0.515463918	Inf	1	1	0.708891947
21	30131500	30150500	loss	.	intergenic	21q21.3	0	1	0.515463918	Inf	1	1	0.708891947
21	38720000	38722000	gain	.	intergenic	21q22.13	0	1	0.515463	Inf	1	1	0.708891

									918				947
21	41649000	41654000	loss	DSCAM	intronic	21q22.2	0	1	0.51 5463 918	Inf	1	1	0.70 8891 947
22	28486000	28486500	gain	TTC28	intronic	22q12.1	0	1	0.51 5463 918	Inf	1	1	0.70 8891 947
22	30252500	30380500	loss	MTMR3	exonic	22q12.2	0	1	0.51 5463 918	Inf	1	1	0.70 8891 947
22	34716500	34717000	gain	.	intergenic	22q12.3	0	1	0.51 5463	Inf	1	1	0.70 8891

									918				947
22	35836500	35844500	loss	.	intergenic	22q12.3	0	1	0.51 5463 918	Inf	1	1	0.70 8891 947
22	38262500	38287500	loss	EIF3L	exonic	22q13.1	0	1	0.51 5463 918	Inf	1	1	0.70 8891 947
22	47146000	47148500	gain	.	intergenic	22q13.3 1	0	1	0.51 5463 918	Inf	1	1	0.70 8891 947
22	48255000	48255500	gain	.	intergenic	22q13.3 1	0	1	0.51 5463	Inf	1	1	0.70 8891

									918				947
3	102025000	102072500	loss	.	intergenic	3q12.3	0	1	0.51 5463 918	Inf	1	1	0.70 8891 947
3	104908000	104933000	gain	.	intergenic	3q13.11	0	1	0.51 5463 918	Inf	1	1	0.70 8891 947
3	116834000	116845000	loss	.	intergenic	3q13.31	0	1	0.51 5463 918	Inf	1	1	0.70 8891 947
3	29922000	29998500	loss	RBMS3	exonic	3p24.1	0	1	0.51 5463	Inf	1	1	0.70 8891

									918				947
3	38852500	38889000	loss	SCN11A	exonic	3p22.2	0	1	0.515463918	Inf	1	1	0.708891947
3	39610500	39655500	gain	.	intergenic	3p22.1	0	1	0.515463918	Inf	1	1	0.708891947
3	79234000	79251500	loss	ROBO1	intronic	3p12.3	0	1	0.515463918	Inf	1	1	0.708891947
3	86353000	86375500	loss	.	intergenic	3p12.1	0	1	0.515463	Inf	1	1	0.708891

									918				947
3	86687500	86724000	loss	.	intergenic	3p12.1	0	1	0.51<br>5463<br>918	Inf	1	1	0.70<br>8891<br>947
4	100964000	101102500	loss	.	intergenic	4q24-q2<br>3	0	1	0.51<br>5463<br>918	Inf	1	1	0.70<br>8891<br>947
4	106968500	106988000	loss	TBCK	intronic	4q24	0	1	0.51<br>5463<br>918	Inf	1	1	0.70<br>8891<br>947
4	111868000	111884000	loss	.	intergenic	4q25	0	1	0.51<br>5463	Inf	1	1	0.70<br>8891

									918				947
4	132294500	133607500	loss	.	intergenic	4q28.3	0	1	0.51<br>5463<br>918	Inf	1	1	0.70<br>8891<br>947
4	145391500	145403000	loss	.	intergenic	4q31.21	0	1	0.51<br>5463<br>918	Inf	1	1	0.70<br>8891<br>947
4	147836500	147846000	loss	TTC29	intronic	4q31.22	0	1	0.51<br>5463<br>918	Inf	1	1	0.70<br>8891<br>947
4	150598500	150608500	loss	.	intergenic	4q31.23	0	1	0.51<br>5463	Inf	1	1	0.70<br>8891

									918				947
4	161562500	161583500	gain	.	intergenic	4q32.1	0	1	0.51 5463 918	Inf	1	1	0.70 8891 947
4	163352000	163404000	loss	.	intergenic	4q32.2	0	1	0.51 5463 918	Inf	1	1	0.70 8891 947
4	164300000	164311500	loss	.	intergenic	4q32.2	0	1	0.51 5463 918	Inf	1	1	0.70 8891 947
4	175383500	175409000	loss	.	intergenic	4q34.1	0	1	0.51 5463	Inf	1	1	0.70 8891

									918				947
4	18735500	18761000	loss	.	intergenic	4p15.31	0	1	0.51 5463 918	Inf	1	1	0.70 8891 947
4	43767000	43782500	gain	.	intergenic	4p13	0	1	0.51 5463 918	Inf	1	1	0.70 8891 947
4	67771500	67780000	loss	.	intergenic	4q13.2	0	1	0.51 5463 918	Inf	1	1	0.70 8891 947
4	70864500	70877000	loss	STATH	exonic	4q13.3	0	1	0.51 5463	Inf	1	1	0.70 8891

									918				947
4	97926500	97952500	loss	.	intergenic	4q22.3	0	1	0.51 5463 918	Inf	1	1	0.70 8891 947
4	98202000	98216000	loss	.	intergenic	4q22.3	0	1	0.51 5463 918	Inf	1	1	0.70 8891 947
4	99540500	99620000	gain	TSPAN5	exonic	4q23	0	1	0.51 5463 918	Inf	1	1	0.70 8891 947
5	102776000	102893500	loss	LOC10246 7212,NUD	exonic	5q21.2-q21.1	0	1	0.51 5463	Inf	1	1	0.70 8891

				T12					918				947
5	108941500	109001500	loss	.	intergenic	5q21.3	0	1	0.51 5463 918	Inf	1	1	0.70 8891 947
5	113432000	113502000	loss	.	intergenic	5q22.3	0	1	0.51 5463 918	Inf	1	1	0.70 8891 947
5	123084500	123089500	loss	.	intergenic	5q23.2	0	1	0.51 5463 918	Inf	1	1	0.70 8891 947
5	136032500	136044500	gain	.	intergenic	5q31.1	0	1	0.51 5463	Inf	1	1	0.70 8891

									918				947
5	141265500	141274000	loss	.	intergenic	5q31.3	0	1	0.515463918	Inf	1	1	0.708891947
5	154738000	154896000	loss	.	intergenic	5q33.2	0	1	0.515463918	Inf	1	1	0.708891947
5	2687500	2700500	loss	.	intergenic	5p15.33	0	1	0.515463918	Inf	1	1	0.708891947
5	43856000	43898000	loss	.	intergenic	5p12	0	1	0.515463	Inf	1	1	0.708891

									918				947
5	44344000	44363000	loss	FGF10	intronic	5p12	0	1	0.51 5463 918	Inf	1	1	0.70 8891 947
6	117754500	117761000	gain	.	intergenic	6q22.1	0	1	0.51 5463 918	Inf	1	1	0.70 8891 947
6	129108500	129173500	loss	.	intergenic	6q22.33	0	1	0.51 5463 918	Inf	1	1	0.70 8891 947
6	14014500	14023500	gain	.	intergenic	6p23	0	1	0.51 5463	Inf	1	1	0.70 8891

									918				947
6	159367500	159368000	gain	.	intergenic	6q25.3	0	1	0.515463918	Inf	1	1	0.708891947
6	24358500	24359500	loss	KAAG1,DCDC2	UTR3	6p22.3	0	1	0.515463918	Inf	1	1	0.708891947
6	3157000	3224500	loss	TUBB2B,TUBB2A,RP1-40E16.9	exonic	6p25.2	0	1	0.515463918	Inf	1	1	0.708891947
6	69421500	69446500	loss	BAI3	intronic	6q12	0	1	0.515463	Inf	1	1	0.708891

									918				947
6	77213000	77313000	loss	.	intergenic	6q14.1	0	1	0.51<br>5463<br>918	Inf	1	1	0.70<br>8891<br>947
6	83327500	83640000	loss	UBE3D	exonic	6q14.1	0	1	0.51<br>5463<br>918	Inf	1	1	0.70<br>8891<br>947
6	86428000	86467000	loss	.	intergenic	6q14.3	0	1	0.51<br>5463<br>918	Inf	1	1	0.70<br>8891<br>947
6	97301500	97308500	gain	.	intergenic	6q16.1	0	1	0.51<br>5463	Inf	1	1	0.70<br>8891

									918				947
6	9778000	9778500	gain	.	intergenic	6p24.3	0	1	0.51 5463 918	Inf	1	1	0.70 8891 947
7	151010000	151074500	gain	NUB1	exonic	7q36.1	0	1	0.51 5463 918	Inf	1	1	0.70 8891 947
7	2775500	2783500	loss	GNA12	intronic	7p22.3	0	1	0.51 5463 918	Inf	1	1	0.70 8891 947
7	69822500	69850500	loss	AUTS2	intronic	7q11.22	0	1	0.51 5463	Inf	1	1	0.70 8891

									918				947
7	80716500	80730500	loss	.	intergenic	7q21.11	0	1	0.51 5463 918	Inf	1	1	0.70 8891 947
7	85069000	85110500	loss	.	intergenic	7q21.11	0	1	0.51 5463 918	Inf	1	1	0.70 8891 947
7	85319500	85334000	loss	.	intergenic	7q21.11	0	1	0.51 5463 918	Inf	1	1	0.70 8891 947
7	93067500	93488000	loss	MIR4652, MIR489,C	exonic	7q21.3	0	1	0.51 5463	Inf	1	1	0.70 8891

				ALCR,MIR653					918				947
7	9445000	9466500	gain	.	intergenic	7p21.3	0	1	0.515463918	Inf	1	1	0.708891947
7	99431000	99434000	gain	CYP3A43	intronic	7q22.1	0	1	0.515463918	Inf	1	1	0.708891947
8	104599500	104608000	loss	RIMS2	intronic	8q22.3	0	1	0.515463918	Inf	1	1	0.708891947
8	114328000	114344000	gain	CSMD3	intronic	8q23.3	0	1	0.51		1	1	0.70

									5463<br>918	Inf			8891<br>947
8	114397000	114441000	loss	CSMD3	intronic	8q23.3	0	1	0.51<br>5463<br>918	Inf	1	1	0.70<br>8891<br>947
8	121054500	121069000	loss	DEPTOR	exonic	8q24.12	0	1	0.51<br>5463<br>918	Inf	1	1	0.70<br>8891<br>947
8	124083000	124087500	gain	TBC1D31	exonic	8q24.13	0	1	0.51<br>5463<br>918	Inf	1	1	0.70<br>8891<br>947
8	125485000	125489500	gain	RNF139-A	exonic	8q24.13	0	1	0.51		1	1	0.70

				S1,RNF139					5463<br>918	Inf			8891<br>947
8	37930500	37938000	loss	.	intergenic	8p11.23	0	1	0.51<br>5463<br>918	Inf	1	1	0.70<br>8891<br>947
8	51644500	51690000	loss	SNTG1	exonic	8q11.21	0	1	0.51<br>5463<br>918	Inf	1	1	0.70<br>8891<br>947
8	83204500	83238500	loss	.	intergenic	8q21.13	0	1	0.51<br>5463<br>918	Inf	1	1	0.70<br>8891<br>947
8	89101500	89152500	loss	MMP16	exonic	8q21.3	0	1	0.51		1	1	0.70

									5463<br>918	Inf			8891<br>947
8	9110500	9244000	loss	RP11-115J<br>16.1	ncRNA_ex<br>onic	8p23.1	0	1	0.51<br>5463<br>918	Inf	1	1	0.70<br>8891<br>947
8	94222000	94336500	loss	.	intergenic	8q22.1	0	1	0.51<br>5463<br>918	Inf	1	1	0.70<br>8891<br>947
8	98410500	98425500	loss	.	intergenic	8q22.1	0	1	0.51<br>5463<br>918	Inf	1	1	0.70<br>8891<br>947
9	103519000	103544000	loss	.	intergenic	9q31.1	0	1	0.51		1	1	0.70

									5463 918	Inf			8891 947
9	128655500	128669500	loss	PBX3	intronic	9q33.3	0	1	0.51 5463 918	Inf	1	1	0.70 8891 947
9	22889500	22924000	loss	.	intergenic	9p21.3	0	1	0.51 5463 918	Inf	1	1	0.70 8891 947
9	80391500	80397000	loss	GNAQ	intronic	9q21.2	0	1	0.51 5463 918	Inf	1	1	0.70 8891 947
10	24119000	24206000	loss	KIAA1217	intronic	10p12.2	1	2	0.52	1.89	1	1	0.70

									3309 385	E+0 0			8891 947
11	35244500	35259500	gain	CD44	exonic	11p13	1	2	0.52 3309 385	1.89 E+0 0	1	1	0.70 8891 947
11	90926000	90968000	loss	.	intergenic	11q14.3	1	2	0.52 3309 385	1.89 E+0 0	1	1	0.70 8891 947
14	58667500	58700000	loss	ACTR10	exonic	14q23.1	1	2	0.52 3309 385	1.89 E+0 0	1	1	0.70 8891 947
16	61622000	61900500	loss	CDH8	exonic	16q21	1	2	0.52	1.89	1	1	0.70

									3309<br>385	E+0<br>0			8891<br>947
17	51982500	52011500	loss	.	intergenic	17q22	1	2	0.52<br>3309<br>385	1.89<br>E+0<br>0	1	1	0.70<br>8891<br>947
17	69584000	69651000	loss	.	intergenic	17q24.3	1	2	0.52<br>3309<br>385	1.89<br>E+0<br>0	1	1	0.70<br>8891<br>947
18	26367500	26399500	loss	.	intergenic	18q12.1	1	2	0.52<br>3309<br>385	1.89<br>E+0<br>0	1	1	0.70<br>8891<br>947
18	36671500	36678000	gain	.	intergenic	18q12.2	1	2	0.52	1.89	1	1	0.70

									3309<br>385	E+0<br>0			8891<br>947
2	195854500	196080500	loss	.	intergenic	2q32.3	1	2	0.52<br>3309<br>385	1.89<br>E+0<br>0	1	1	0.70<br>8891<br>947
20	7255500	7381000	loss	MIR8062	ncRNA_ex<br>onic	20p12.3	1	2	0.52<br>3309<br>385	1.89<br>E+0<br>0	1	1	0.70<br>8891<br>947
21	19396500	19429000	loss	CHODL	UTR5	21q21.1	1	2	0.52<br>3309<br>385	1.89<br>E+0<br>0	1	1	0.70<br>8891<br>947
4	58665500	58728000	loss	.	intergenic	4q12	1	2	0.52	1.89	1	1	0.70

									3309385	E+00			8891947
6	82407500	82434000	loss	.	intergenic	6q14.1	1	2	0.523309385	1.89E+00	1	1	0.708891947
8	60633500	60661000	loss	.	intergenic	8q12.1	1	2	0.523309385	1.89E+00	1	1	0.708891947
8	65113000	65248500	loss	.	intergenic	8q12.3	1	2	0.523309385	1.89E+00	1	1	0.708891947
9	83164500	83237000	loss	.	intergenic	9q21.31	1	2	0.52	1.89	1	1	0.70

									3309385	E+00			8891947
19	32289000	32289500	gain	.	intergenic	19q12	2	3	0.529280718	1.42E+00	1	1	0.712428649
20	58434500	58470500	loss	SYCP2	exonic	20q13.33	2	3	0.529280718	1.42E+00	1	1	0.712428649
12	82103500	82144500	loss	PPFIA2	intronic	12q21.31	2	2	0.712401586	9.39E-01	1	1	0.92593734
5	85646500	85717500	loss	.	intergenic	5q14.3	2	2	0.71	9.39	1	1	0.92

									2401586	E-01			593734
11	27460500	27619000	gain	BDNF-AS, LIN7C,MIR8087,LGR4	exonic	11p14.1	1	1	0.766518883	9.40 E-01	1	1	0.92593734
11	46589500	46606500	loss	AMBRA1	intronic	11p11.2	1	1	0.766518883	9.40 E-01	1	1	0.92593734
12	43433000	43526000	loss	.	intergenic	12q12	1	1	0.766518883	9.40 E-01	1	1	0.92593734

12	44724500	44873000	loss	TMEM117	exonic	12q12	1	1	0.766518883	9.40E-01	1	1	0.92593734
12	84768000	84925000	loss	.	intergenic	12q21.31	1	1	0.766518883	9.40E-01	1	1	0.92593734
13	38329000	38343500	loss	TRPC4	intronic	13q13.3	1	1	0.766518883	9.40E-01	1	1	0.92593734
13	68279500	68337000	loss	.	intergenic	13q21.32	1	1	0.766518883	9.40E-01	1	1	0.92593734

13	78749000	78912500	loss	RNF219-AS1	ncRNA_exonic	13q22.3	1	1	0.766518883	9.40E-01	1	1	0.92593734
14	49660500	49694000	loss	.	intergenic	14q21.3	1	1	0.766518883	9.40E-01	1	1	0.92593734
14	78279000	78284000	loss	ADCK1	intronic	14q24.3	1	1	0.766518883	9.40E-01	1	1	0.92593734
15	35896500	35905500	loss	DPH6-AS1	ncRNA_intronic	15q14	1	1	0.766518883	9.40E-01	1	1	0.92593734

15	47709000	47762500	loss	SEMA6D	intronic	15q21.1	1	1	0.76 6518 883	9.40 E-01	1	1	0.92 5937 34
15	49931500	49975000	loss	DTWD1	exonic	15q21.2	1	1	0.76 6518 883	9.40 E-01	1	1	0.92 5937 34
16	59446000	59489000	loss	.	intergenic	16q21	1	1	0.76 6518 883	9.40 E-01	1	1	0.92 5937 34
17	68776000	68826000	loss	.	intergenic	17q24.3	1	1	0.76 6518 883	9.40 E-01	1	1	0.92 5937 34

18	38064500	38068500	gain	.	intergenic	18q12.3	1	1	0.766518883	9.40E-01	1	1	0.92593734
2	140347000	140391000	loss	.	intergenic	2q22.1	1	1	0.766518883	9.40E-01	1	1	0.92593734
2	81164000	81250000	gain	.	intergenic	2p12	1	1	0.766518883	9.40E-01	1	1	0.92593734
21	16263000	16265500	loss	.	intergenic	21q11.2	1	1	0.766518883	9.40E-01	1	1	0.92593734

21	35012500	35015000	gain	CRYZL1,ITSN1	UTR5	21q22.11	1	1	0.766518883	9.40E-01	1	1	0.92593734
22	27308000	28286000	gain	RP1-90L6.2,MN1,PITPNB	exonic	22q12.1	1	1	0.766518883	9.40E-01	1	1	0.92593734
3	30204500	30501000	loss	.	intergenic	3p24.1	1	1	0.766518883	9.40E-01	1	1	0.92593734
4	21270000	21418500	gain	KCNIP4	exonic	4p15.2-p15.31	1	1	0.766518883	9.40E-01	1	1	0.92593734

4	93877500	93987500	loss	GRID2	intronic	4q22.2	1	1	0.766518883	9.40E-01	1	1	0.92593734
5	30579500	30765000	loss	.	intergenic	5p13.3	1	1	0.766518883	9.40E-01	1	1	0.92593734
5	89011000	89032000	loss	.	intergenic	5q14.3	1	1	0.766518883	9.40E-01	1	1	0.92593734
6	1652500	1653000	gain	GMDS	intronic	6p25.3	1	1	0.766518883	9.40E-01	1	1	0.92593734

6	82720000	82745500	loss	.	intergenic	6q14.1	1	1	0.766518883	9.40E-01	1	1	0.92593734
6	98722500	98943500	loss	.	intergenic	6q16.1	1	1	0.766518883	9.40E-01	1	1	0.92593734
9	107828000	107838000	loss	.	intergenic	9q31.1	1	1	0.766518883	9.40E-01	1	1	0.92593734
9	113404500	113503000	loss	MUSK	exonic	9q31.3	1	1	0.766518883	9.40E-01	1	1	0.92593734

9	24015000	24067000	loss	.	intergenic	9p21.3	1	1	0.766518883	9.40E-01	1	1	0.92593734
9	2589500	2901000	gain	VLDLR-AS1,KCNV2,KIAA0020,VLDLR	exonic	9p24.2	1	1	0.766518883	9.40E-01	1	1	0.92593734
9	85402500	85460500	loss	.	intergenic	9q21.32	1	1	0.766518883	9.40E-01	1	1	0.92593734
16	62544000	62600000	loss	.	intergenic	16q21	4	3	0.802434	6.97E-01	1	1	0.966569

									81				203
20	32911000	33007500	loss	ITCH	exonic	20q11.22	3	2	0.834482165	6.21E-01	1	1	1
13	103380500	103403000	gain	CCDC168	exonic	13q33.1	5	3	0.879855066	5.52E-01	1	1	1
3	33004500	33015500	gain	.	intergenic	3p22.3	5	3	0.879855066	5.52E-01	1	1	1
11	66840000	67006000	loss	KDM2A	exonic	11q13.2	2	1	0.888123	4.66E-01	1	1	1

									631				
11	87808500	87844000	loss	.	intergenic	11q14.2	2	1	0.88 8123 631	4.66 E-01	1	1	1
11	95301500	95337000	loss	.	intergenic	11q21	2	1	0.88 8123 631	4.66 E-01	1	1	1
12	49710500	49723000	loss	TROAP	exonic	12q13.1 2	2	1	0.88 8123 631	4.66 E-01	1	1	1
17	21176000	21220000	gain	MAP2K3	exonic	17p11.2	2	1	0.88 8123	4.66 E-01	1	1	1

									631				
18	21895500	21896000	gain	OSBPL1A	intronic	18q11.2	2	1	0.88 8123 631	4.66 E-01	1	1	1
18	50514500	50664500	loss	DCC	exonic	18q21.2	2	1	0.88 8123 631	4.66 E-01	1	1	1
18	50730500	50740500	loss	DCC	exonic	18q21.2	2	1	0.88 8123 631	4.66 E-01	1	1	1
18	50833500	50839500	loss	DCC	intronic	18q21.2	2	1	0.88 8123	4.66 E-01	1	1	1

									631				
4	165173500	165209000	loss	1-Mar	intronic	4q32.3	2	1	0.88 8123 631	4.66 E-01	1	1	1
17	27078000	27206000	loss	ERAL1,MI R4732,MI R451A,MI R451B,FA M222B,MI R144	exonic	17q11.2	17	12	0.91 7826 552	6.19 E-01	1	1	1
11	91556000	91599500	loss	.	intergenic	11q14.3	3	1	0.94 6697	3.08 E-01	1	1	1

									646				
11	91812000	91830500	loss	.	intergenic	11q14.3	3	1	0.94 6697 646	3.08 E-01	1	1	1
12	110340500	110349000	gain	TCHP	exonic	12q24.1 1	3	1	0.94 6697 646	3.08 E-01	1	1	1
14	93619500	93626500	loss	.	intergenic	14q32.1 2	3	1	0.94 6697 646	3.08 E-01	1	1	1
20	48450500	48461500	loss	SLC9A8	exonic	20q13.1 3	3	1	0.94 6697	3.08 E-01	1	1	1

									646				
1	28610500	29081500	loss	GMEB1,RCC1,MED18,TAF12,SNORA16A,SNORA61,SNORA44,RAB42,SNHG12,SNORD99,TRNAU1AP,RNU11,Y	exonic	1p35.3	4	1	0.974751517	2.29E-01	1	1	1

				THDF2,SNHG3,PHACTR4									
1	31502500	31652000	loss	PUM1	exonic	1p35.2	4	1	0.974751517	2.29E-01	1	1	1
21	23089000	23091000	loss	.	intergenic	21q21.1	4	1	0.974751517	2.29E-01	1	1	1
5	132239500	132383000	loss	AFF4,ZCCHC10	exonic	5q31.1	4	1	0.974751517	2.29E-01	1	1	1

7	4584000	4878500	gain	MIR4656, AP5Z1,FOXK1,RADIL	exonic	7p22.1	4	1	0.974751517	2.29E-01	1	1	1
17	30711500	30761500	loss	.	intergenic	17q11.2	10	4	0.981755149	3.52E-01	1	1	1
6	134579000	134609000	loss	SGK1	intronic	6q23.2	18	9	0.988204207	4.19E-01	1	1	1
12	122329500	122414500	gain	PSMD9,WDR66	exonic	12q24.31	5	1	0.988110	1.81E-01	1	1	1

									503				
21	28172500	28191500	loss	.	intergenic	21q21.3	5	1	0.98 8110 503	1.81 E-01	1	1	1
22	50759500	50853000	loss	DENND6B ,PPP6R2	exonic	22q13.3 3	13	5	0.99 1815 448	3.30 E-01	1	1	1
12	50971000	50977500	loss	DIP2B	intronic	12q13.1 2	6	1	0.99 4434 703	1.49 E-01	1	1	1
15	25362500	25407000	loss	IPW,PWA R1	ncRNA_ex onic	15q11.2	6	1	0.99 4434	1.49 E-01	1	1	1

									703				
2	59635500	59654000	gain	.	intergenic	2p16.1	6	1	0.99 4434 703	1.49 E-01	1	1	1
20	35526500	35786000	loss	MROH8,S AMHD1,R BL1	exonic	20q11.2 3	6	1	0.99 4434 703	1.49 E-01	1	1	1
12	113739000	114706000	gain	RP11-438 N16.1,SDS ,RBM19,L HX5,PLBD 2,SLC8B1,	exonic	12q24.1 3	9	2	0.99 6460 576	1.94 E-01	1	1	1

				SDSL									
20	47804000	47845500	loss	DDX27,STAU1	exonic	20q13.13	9	2	0.996460576	1.94E-01	1	1	1
16	11473500	11569000	loss	.	intergenic	16p13.13	7	1	0.997410798	1.27E-01	1	1	1
17	37396000	37679500	loss	MED1,CDK12,FBXL20	exonic	17q12	17	6	0.997963265	2.91E-01	1	1	1
22	41245500	41263000	gain	DNAJB7,XPNPEP3,S	exonic	22q13.2	8	1	0.998802	1.10E-01	1	1	1

				T13					842				
3	48728000	49003500	loss	ARIH2OS,<br>SLC25A20<br>,ARIH2,IP<br>6K2,PRKA<br>R2A,PRK<br>AR2A-AS1	exonic	3p21.31	8	1	0.99<br>8802<br>842	1.10<br>E-01	1	1	1
13	101889500	101896500	loss	NALCN	exonic	13q33.1	11	2	0.99<br>9135<br>184	1.55<br>E-01	1	1	1
15	41343500	41754000	loss	RTF1,NUS<br>AP1,CHP1,	exonic	15q15.1	9	1	0.99<br>9449	9.63<br>E-02	1	1	1

				INO80,OIP5-AS1,OIP5,EXD1,NDUFAF1					954				
17	30187500	30328000	loss	SUZ12,UTP6	exonic	17q11.2	10	1	0.999748892	8.57E-02	1	1	1
17	35653500	35835500	loss	TADA2A,ACACA,C17orf78	exonic	17q12	10	1	0.999748892	8.57E-02	1	1	1
19	47715500	47905500	loss	C5AR1,CCDC9,C5AR	exonic	19q13.32	10	1	0.999748	8.57E-02	1	1	1

				2,BBC3,MIR3190,DHX34,PRR24,MIR3191					892				
5	127403000	127414000	loss	LINC01184	ncRNA_intronic	5q23.3	10	1	0.999748892	8.57E-02	1	1	1
5	29319000	29530000	gain	LOC101929681	ncRNA_exonic	5p13.3	11	1	0.99988611	7.70E-02	1	1	1
7	73656500	74094500	loss	GTF2IRD1,CLIP2,RF	exonic	7q11.23	11	1	0.999886	7.70E-02	1	1	1

				C2,GTF2I					11			
19	14847500	14848000	gain	EMR2	intronic	19p13.12	14	2	0.999904269	1.18E-01	1	1	1
19	4724000	4952500	loss	ARRDC5,FEM1A,PLIN3,UHRF1,MIR7-3HG,TICAM1,MIR7-3,MIR4747	exonic	19p13.3	12	1	0.999948687	6.97E-02	1	1	1
22	34863500	34865000	loss	.	intergenic	22q12.3	46	19	0.99	2.47	1	1	1

									9998<br>06	E-01			
13	99252000	99297500	loss	.	intergenic	13q32.2	17	1	0.99<br>9999<br>141	4.63<br>E-02	1	1	1
17	37085500	37155500	loss	FBXO47,L<br>INC00672	exonic	17q12	17	1	0.99<br>9999<br>141	4.63<br>E-02	1	1	1
19	36066500	36068500	gain	.	intergenic	19q13.1<br>2	27	5	0.99<br>9999<br>312	1.32<br>E-01	1	1	1
11	48977500	50203500	gain	OR4C12,F	exonic	11p11.1	20	1	0.99	3.78	1	1	1

				OLH1,LOC440040,TRIM49B,TRIM64C,OR4C13		2			9999932	E-02			
17	1226500	1244500	loss	.	intergenic	17p13.3	20	1	0.999999932	3.78E-02	1	1	1
4	71485000	71493500	loss	.	intergenic	4q13.3	21	1	0.999999972	3.55E-02	1	1	1
11	63183000	63216000	gain	MIR3680-2	ncRNA_int	11q12.3	39	6	1	9.11	1	1	1

				,MIR3680-1	ronic					E-02			
12	0	169500	gain	FAM138D,LOC100288778	intergenic	12p13.33	94	57	1	0.00E+00	1	1	1
10	38519000	39154500	gain	ACTR3BP5,LINC00999,HSD17B7P2,SEPT7P9	ncRNA_exonic	10p11.1	58	9	1	6.24E-02	1	1	1
X	538000	2128000	loss	IL3RA,SLC25A6,AS	exonic	Xp22.33	94	5	1	0	1	1	1

				MT,ASMTL,SHOX,CSF2RA,AKAP17A,ASMTL-AS1,P2RY8,CRLF2,MIR3690-2,MIR3690									
21	22653000	22678000	loss	RNU6-67P,NCAM2	exonic	21q21.1	45	3	1	3.42E-02	1	1	1
1	142535500	145118000	gain	FAM72C,L	intergenic	1q12	93	1	1	1.78	1	1	1

				INC00623, PFN1P2,PPIAL4B,PPIAL4G,RP6-206I17.2, RP11-640M9.1,MIR6077-2,NBPF9,PPIAL4C,SEC22B,MIR6077-1,LOC65						E-04			

				3513,PPIAL4A,NBPF20,LINC01138,LINC00875,ANKRD20A12P,NBPF8,PDE4DIP,FAM72D									
12	90485500	90504000	loss	.	intergenic	12q21.33	45	1	1	1.12E-02	1	1	1
14	34936500	35179500	loss	SNX6,EAP	exonic	14q13.1	44	1	1	1.17	1	1	1

				P						E-02				
14	74284000	74345500	loss	PTGR2	exonic	14q24.3	51	1	1	8.70 E-03	1	1	1	
20	42266500	42274500	loss	IFT52	exonic	20q13.1 2	41	1	1	1.33 E-02	1	1	1	
20	52115000	52118000	loss	.	intergenic	20q13.2	47	1	1	1.03 E-02	1	1	1	
21	22817500	22849500	loss	NCAM2	exonic	21q21.1	45	1	1	1.12 E-02	1	1	1	
21	22971500	23001000	loss	.	intergenic	21q21.1	45	1	1	1.12 E-02	1	1	1	
22	39342500	39364500	gain	APOBEC3	exonic	22q13.1	54	1	1	7.66	1	1	1	

				A,APOBE C3A_B						E-03			
5	83948500	84105000	loss	.	intergenic	5q14.3	82	1	1	1.59 E-03	1	1	1
X	283500	335500	gain	PPP2R3B	exonic	Xp22.3 3	94	1	1	0	1	1	1

CNV burden analysis identified a total of 433 CNVs and 17 of them were significantly associated with sporadic TAD (P<0.05).

Supplementary Table 2. TAD Affected individuals with CNVs in DSCAM, APP, LINC00907, and PROCR.

CHR	Start	End	CNV Type	Gene	CytoBand	Case_shared No.	Patient ID
21	41702000	41702500	Loss	*DSCAM*	21q22.2	13	A64, A125, A162, A217, A234, A280, A281,

							A313, A340, A342, A343, A436, A484
21	27379500	27380500	Loss	*APP*	21q21.3	8	A125, A162, A234, A295, A343, A438, A451, A483
18	40153000	40174500	Loss	*LINC00907*	18q12.3	6	A131, A168, A180, A199, A287, A321
20	33754500	33764500	Gain	*PROCR*	20q11.22	5	A69, A125, A199, A282, A483

Supplementary Table 3. Expression Profiles of the Four Candidate Genes.

Gene	Chr	Molecular function	Microarray Fold change	P value
APP	21	Functions as a cell surface receptor and performs physiological functions on the surface of neurons relevant to neurite growth, neuronal adhesion and axonogenesis. Involved in cell mobility and transcription regulation through protein-protein interactions.	0.61	0.032048

DSCAM	21	Neural cell adhesion molecule in the Down syndrome critical region, with several transcripts, largely expressed in the developing nervous system and adult brain, immunoglobulin superfamily; Potentially involved in congenital heart disease; Mediates intracellular signaling by stimulating the activation of MAPK8 and MAP kinase p38.	0.74	0.792942
LINC00907	18	No data available.	NA	NA
PROCR	20	Binds activated protein C. Enhances protein C activation by the thrombin-thrombomodulin complex; plays a role in the protein C pathway controlling blood coagulation.	2.12	0.059154

Chr, chromosome; SD, standard deviation.

Supplementary Table 4. The probes and universal primers used in MLPA

Oligo name	Oligo Sequence
**L-universal primers**	5’- GGGTTCCCTAAGGGTTGGA -3’
**R-universal primers**	5’- TCTAGATTGGATCTTGCTGGCAC -3’
**DC-LPO-1**	GGGTTCCCTAAGGGTTGGAGCATTCTTTCATAATGAAACCAATCTTACACATAGTCTTTTTTTCCCTCCAATTA
**DC-RPO-1**	GGGTAAAGTAATTTGAGATATCGCTTATAAAACACTTAATAAAATGCCATATTTCTAGATTGGATCTTGCTGGCAC
**DC-LPO-2**	GGGTTCCCTAAGGGTTGGATTGTAAATACATATTATTTTATTTACAATGTAAAAAATCATGTATATACATTT
**DC-RPO-2**	AAAATCAATATGTAATGTAATATATGCAATACATAACATATATTTATGCCTCTAGATTGGATCTTGCTGGCAC
**AP-LPO-1**	GGGTTCCCTAAGGGTTGGAGTAGAGTAAAAAGGGCAAATGTAAGACAAATATAATTTATTTTTGTAA

**AP-RPO-1**	TAAGTTACTTCCTTAATGTAACAGTGGAATAAAATGAAAACGACATCTAGATTGGATCTTGCTGGCAC
**AP-LPO-2**	GGGTTCCCTAAGGGTTGGATTGTTATTGCTGTAATTCTAAGTATTACTACGTGGTGACATTT
**AP-RPO-2**	CATGAGCCTAAGTTATTAAAATCAAACTTGTATTCTGTTGTCTAGATTGGATCTTGCTGGCAC
**LI-LPO-1**	GGGTTCCCTAAGGGTTGGATTGTGTGAGCTAAAAAGCTATGTCTGTGAGGTCTCT
**LI-RPO-1**	GCATGCAGCTCTGTTGAAGCTAATGGAGTCAATCTAGATTGGATCTTGCTGGCAC
**LI-LPO-2**	GGGTTCCCTAAGGGTTGGATTACCAAGAGGCAAGTCAGGTGGTCTGGGATTG
**LI-RPO-2**	CTGCTTTGCCTCTAGCTGGCTTCAAGTTGATCTAGATTGGATCTTGCTGGCAC
**LI-LPO-3**	GGGTTCCCTAAGGGTTGGATCTGATGGCTGATCCAAAGACACTGTTTCT
**LI-RPO-3**	CAGGAATAGCTGATTGGTTGTCTGCAAATCTAGATTGGATCTTGCTGGCAC
**PR-LPO-1**	GGGTTCCCTAAGGGTTGGATTAGCCAGTTTGCAACAGCTGAGAGGTGAGCATGGAA
**PR-RPO-1**	GCTCTTGCATATATTCAGTTCAGAGAATGGGTGCTTTCTAGATTGGATCTTGCTGGCAC
**PR-LPO-2**	GGGTTCCCTAAGGGTTGGATTTGGAACTGGGGAGAAAGAGATGCTGTGCCTAATAGAACT

**PR-RPO-2**	TATGGGCGATCAGGCTACTGAAGTGGCCCTGTTTAACTCTAGATTGGATCTTGCTGGCAC
**PR-LPO-3**	GGGTTCCCTAAGGGTTGGATCGTCCTGCTGGCATAACCTCTTGGGATA
**PR-RPO-3**	GACCCTGTTGGAAGGCCCTGACACTCTAGATTGGATCTTGCTGGCAC

DC, DSCAM; AP, APP; LI, LINC00907; PR, PROCR
